# Linking Research Data with Physically Preserved Research Materials in Chemistry

**DOI:** 10.1038/s41597-025-04404-2

**Published:** 2025-01-22

**Authors:** Chia-Lin Lin, Pei-Chi Huang, Simone Gräßle, Christoph Grathwol, Pierre Tremouilhac, Sylvia Vanderheiden, Patrick Hodapp, Sonja Herres-Pawlis, Alexander Hoffmann, Fabian Fink, Georg Manolikakes, Till Opatz, Andreas Link, M. Manuel B. Marques, Lena J. Daumann, Manuel Tsotsalas, Frank Biedermann, Hatice Mutlu, Eric Täuscher, Felix Bach, Tim Drees, Steffen Neumann, Shashank S. Harivyasi, Nicole Jung, Stefan Bräse

**Affiliations:** 1 Institute of Biological and Chemical Systems - Functional Molecular Systems (IBCS-FMS), Karlsruhe Institute of Technology, Kaiserstraße 12, 76131 Karlsruhe, Germany; 2 Institute for Biological Interfaces 3 - Soft Matter Laboratory (IBG 3 - SML), Karlsruhe Institute of Technology, Kaiserstraße 12, 76131 Karlsruhe, Germany; 3 RWTH Aachen University, Institute of Inorganic Chemistry, Landoltweg 1a, 52074 Aachen, Germany; 4 RPTU Kaiserslautern-Landau, Department Chemie, Erwin-Schrödinger-Str. Geb. 54, 67663 Kaiserslautern, Germany; 5 JGU Mainz, Department Chemie, Duesbergweg 10-14, 55128 Mainz, Germany; 6 Universität Greifswald, Institut für Pharmazie, Friedrich-Ludwig-Jahn-Str. 17, 17489 Greifswald, Germany; 7 LAQV-REQUIMTE, Department of Chemistry, NOVA School of Science and Technology, Universidade Nova de Lisboa, 2829-516 Caparica, Portugal; 8 Chair of Bioinorganic Chemistry, Heinrich-Heine-Universität Düsseldorf, Universitätsstr. 13, 40225 Düsseldorf, Germany; 9 Institute of Functional Interfaces (IFG), Karlsruhe Institute of Technology, Kaiserstraße 12, 76131 Karlsruhe, Germany; 10 Institute of Nanotechnology (INT), Karlsruhe Institute of Technology, Kaiserstraße 12, 76131 Karlsruhe, Germany; 11 Institut de Science des Matériaux de MulhouseUMR 7361 CNRS/Université de Haute Alsace15 rue Jean Starcky, Mulhouse Cedex, 68057 France; 12 Technische Universität Ilmenau, Institut für Chemie und Biotechnik, Weimarer Straße 25, 98693 Ilmenau, Germany; 13 FIZ Karlsruhe – Leibniz-Institut für Informationsinfrastruktur GmbH, Hermann-von-Helmholtz-Platz 1, 76344 Eggenstein-Leopoldshafen, Germany; 14 Legal Affairs, Karlsruhe Institute of Technology, Kaiserstraße 12, 76131 Karlsruhe, Germany; 15 Leibniz Institute of Plant Biochemistry, Computational Plant Biochemistry group, Halle, Germany; 16 Karlsruhe Nano Micro Facility (KNMFi), Karlsruhe Institute of Technology, Kaiserstraße 12, 76131 Karlsruhe, Germany; 17 Institute of Organic Chemistry (IOC), Karlsruhe Institute of Technology, Kaiserstraße 12, 76131 Karlsruhe, Germany

**Keywords:** Chemistry, Chemical libraries

## Abstract

Results of scientific work in chemistry can usually be obtained in the form of materials and data. A big step towards transparency and reproducibility of the scientific work can be gained if scientists publish their data in research data repositories in a FAIR manner. Nevertheless, in order to make chemistry a sustainable discipline, obtaining FAIR data is insufficient and a comprehensive concept that includes preservation of materials is needed. In order to offer a comprehensive infrastructure to find and access data and materials that were generated in chemistry projects, we combined the infrastructure Chemotion repository with an archive for chemical compounds. Samples play a key role in this concept: we describe how FAIR metadata of a virtual sample representation can be used to refer to a physically available sample in a materials’ archive and to link it with the FAIR research data gained using the said sample. We further describe the measures to make the physically available samples not only FAIR through their metadata but also findable, accessible and reusable.

## Introduction

Sustainable work and provisioning of research results for others is an essential criterion for efficiency in scientific research. Only when results are accessible to the entire scientific community, and thereby reusable, can scientific progress be accelerated in a targeted manner. Since the publication of the FAIR data principles^1^, more and more scientists and associated stakeholders such as funding agencies and journals/publishers support generation and provision of FAIR data across disciplines. Accordingly, data should be Findable, Accessible, Interoperable and Reusable (FAIR), especially the research data that form the basis of publications. In chemistry and materials science, a variety of initiatives promote provision of FAIR data or provide assistance with implementation of FAIR data measures. Established stakeholders that supported the concepts of FAIR data even before their explicit publication include IUPAC^2^, RDA^3^ and CODATA^4^. These have been joined by other important groups such as EOSC^5^ and the National Research Data Infrastructure (NFDI)^6^ ^in Germany^, in particular its consortium for Chemistry (NFDI4Chem)^7^. In future, NFDI4Chem plans to strengthen the chemical community by providing an infrastructure for generating and provisioning FAIR data. Adhering to the FAIR data principles and aligning research processes to obtain FAIR data can form the basis of substantial improvements in data availability and quality. Through transparency, FAIR data can also strengthen trust in research results and promote subsequent usage that is systematic and frictionless.

In synthetic chemistry, the FAIR principles must be extended beyond data and descriptive metadata i.e. synthetic chemists can be encouraged to provide more than data for documentation and subsequent use. In this domain, it is often possible for chemists to substantiate results of reactions in the form of products, and thus provide physical evidence for the research work and its quality. Where the reaction products obtained are stable, they can be collected, stored and registered so that, when suitable, the result can be used directly for further studies. Possible examples of such direct reuse are independent reproduction of experiments, use of these deposited chemical samples for reactions, and analysis of the samples by characterization or screening techniques. Numerous further scenarios are conceivable in which such stored substances could accelerate knowledge gain, especially when they are unambiguously linked to other research output like journal publications and research data. Additionally, such a reusable collection of samples would promote transparency.

Initiatives that provide access to scientific physical collections of materials exist already in other disciplines, such as Geosciences, Microbiology, Botanical Science and Natural History^8^, where collecting and archiving samples for further reuse is widely accepted as an important aspect of scientific work. Examples of such collections of physical samples include the scientific collections of the U.S. Geological Survey^9^ and the collections of drilling cores at the IODP (International Ocean Discovery Program) Core Repository^10^. Some of the physical collections aim to make their samples FAIR, using different concepts to define and establish suitable identifiers that were developed in the past. While many of the currently suggested procedures include the concept of International Generic Sample Number (IGSN)^11,12^ into their processes, others find related solutions to enable a straightforward implementation^13,14^. These different initiatives have developed recommendations that may be a helpful guide to design physical archives that describe materials in a FAIR manner^15,16^.

In chemistry, a few centers worldwide are making efforts to collect and store chemical substances for subsequent use. Exemplary well-known initiatives for the systematic collection of mostly commercial but also partly academic substances are the Compounds Australia^17^, EU-OPENSCREEN^18^, Chimiotheque National (ChemBioFrance)^19^, and the Boston University Center for Molecular Discovery (BU-CMD)^20^. The known initiatives collect and register chemical substances for medical or pharmaceutical application purposes but, as far as we know, do not offer open, general subsequent usage of various kinds.

Thus, the work described in this article was started with the aim to provide for concepts and infrastructure that enable a sustainable model to collect, archive, and reuse the physical results of chemical research work and to connect them with existing research data infrastructure in chemistry.

## Results

### Principles and concept design

**Fig. 1:**
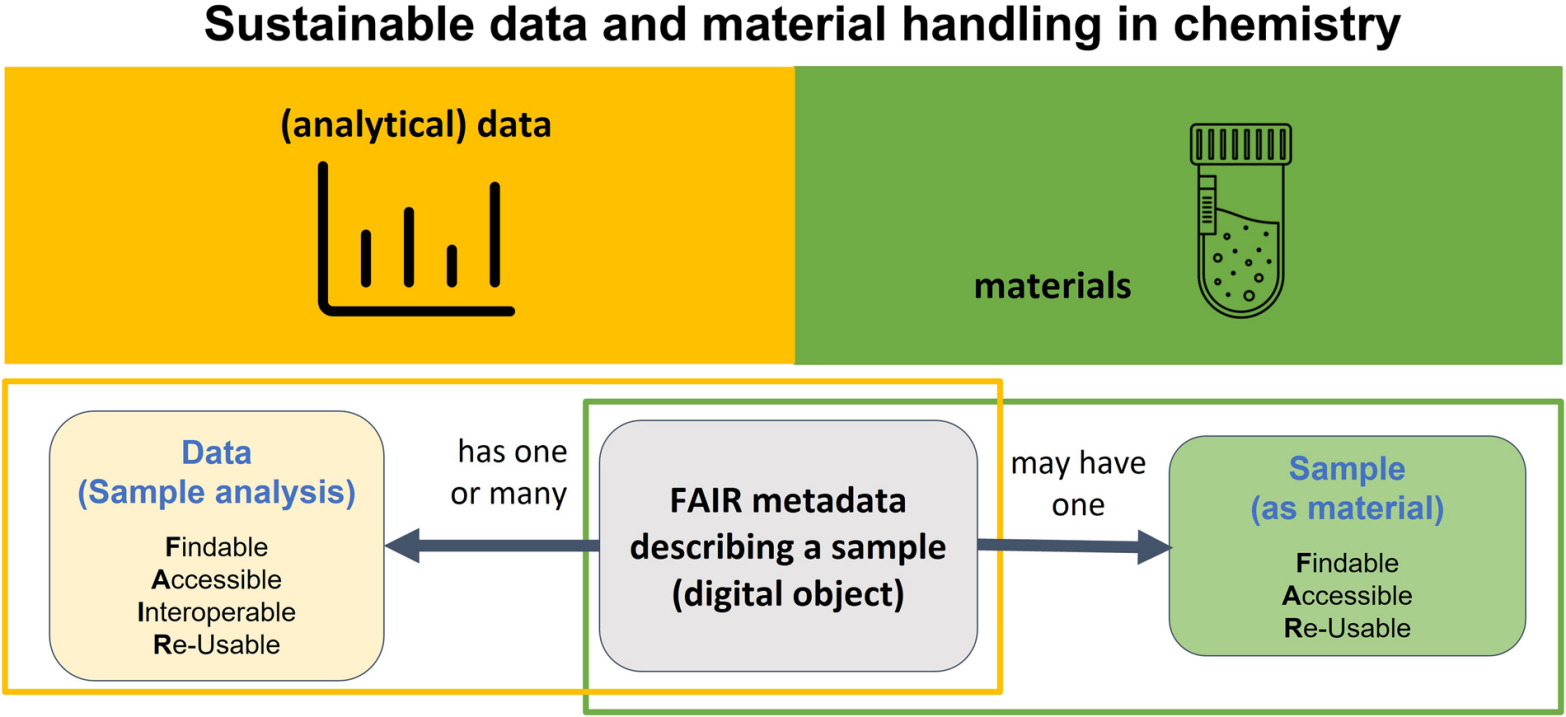
Extending the principles of FAIR data (usually considered in chemistry is the left part in yellow including FAIR metadata for a sample) with a concept for findable, accessible and reusable samples consisting of the same FAIR metadata of a sample and additional measures meeting the requirements of materials’ collection, storage and provision (right part in green). [ref Icons 2: Tube: *Digithrust from the noun project*, Data: *Popular from the noun project*].

Scientific outcome in chemistry consists of data and materials and further studies also often depend on availability of both. Therefore, we suggest complementing the FAIR (meta)data principles with a concept for materials that address sustainable access to physical objects11 such as chemical samples, allowing for reuse, wherever possible. The concept should make it possible to secure research results in their physical form, verify the results obtained and increase reusability of the materials in addition to the reuse of already well-established data. To this aim, chemical compounds or, more precisely, samples of chemical compounds – which are the starting point of analytical studies or the outcome of synthetic studies in chemistry – should be preserved and made available. As a first step, the metadata of samples would need to be **F**indable, **A**ccessible, **I**nteroperable, and **R**eusable (=**FAIR** metadata for samples). As a following step, the samples would need to be registered and stored in a materials’ archive to be Findable, **A**ccessible, and made available under suitable access policies and rules to be physically **R**eusable (=F**AR** material for samples). This concept is further referred to as “FAIR-FAR samples” (Fig. 1).

### The FAIR-FAR sample concept in detail

**Fig. 2:**
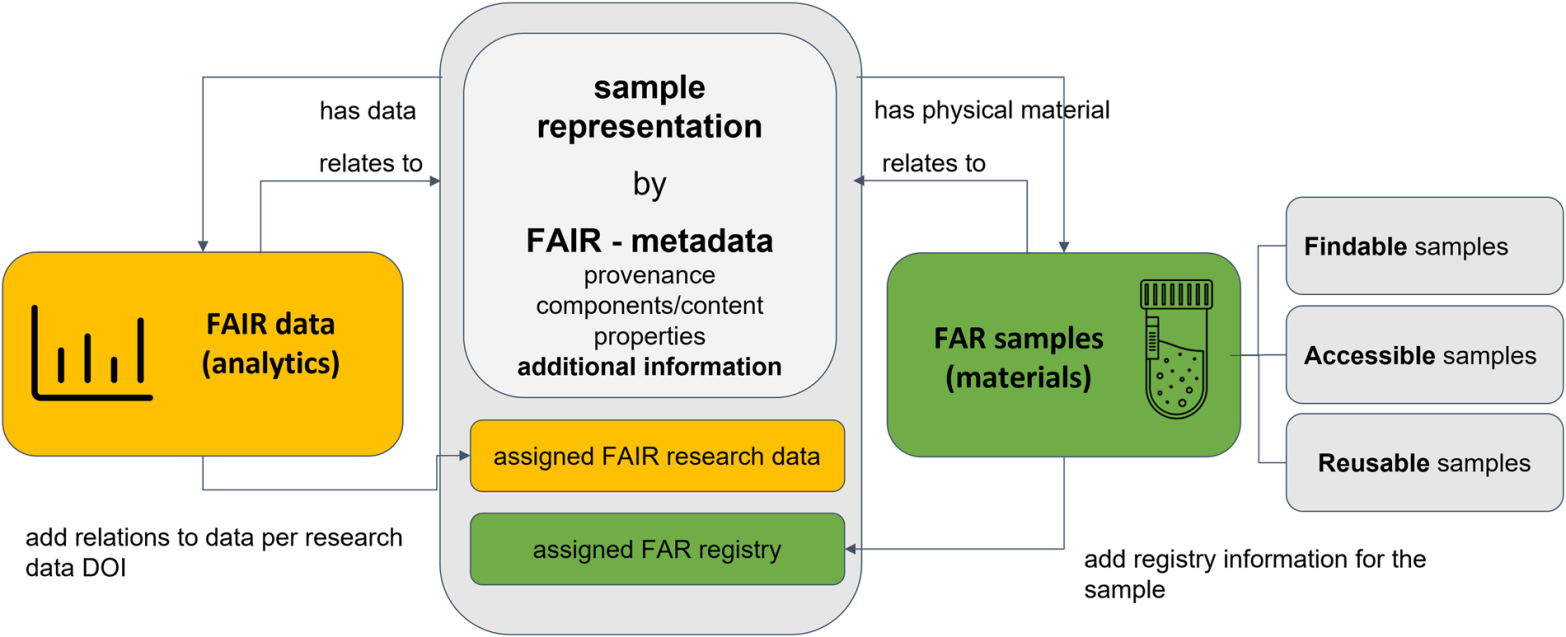
Design of the infrastructure towards more sustainable scientific work in chemistry: The concepts of FAIR data, e.g. analytical measurements, are combined with the concept of findable, accessible and reusable samples through the virtual sample representation given by a sample’s FAIR metadata. [ref Icons 2: Tube: *Digithrust from the noun project*, Data: *Popular from the noun project*].

Metadata for a sample can be seen as a virtual representation of the sample or, in other words, a digital object to which a globally unique and persistent identifier can be assigned. Metadata that are part of such a virtual sample representation include information on the sample’s provenance, components/content, and properties. Most of the metadata assigned to such a virtual sample representation are relevant for a FAIR data approach if it comes to a comprehensive description of analytical measurements of chemical samples. This is because chemistry samples can usually be described with standardized metadata that is referred to by both, the physical sample as well as the data obtained by analyzing it. Efficient concepts that consider the deposition of FAIR research data and the deposit of FAIR-FAR samples could, therefore, closely link both of them. A design to implement this is depicted in Fig. 2, showing a virtual sample representation described by rich standardized metadata, which is complemented by (1) information on further relevant data assigned to the sample (Fig. 2, left), and (2) information on available samples’ location and its unique registry or reference number (Fig. 2, right). The information on the samples’ location must come from the register of samples made available *via* an archive for materials. A standardized process for the stockpiling of the samples, with a suitable validation of the materials and mechanisms to assign unique identifiers, is needed to gain such a deposition and registration of samples. Further, it means that the delivery and sample-sharing process (if applicable) is well described and standardized, including rules that might control access to the materials. Therefore, a sustainable materials approach should include information on the usage conditions of samples, such as legal agreements, and if available, safety information.

#### Implementation in the form of infrastructure

The combined approach for research data and materials/samples makes sense due to the obvious methodical connection of both *via* the virtual sample representation (Fig. 2) and the often observed issue that chemical samples are only efficiently reusable by others if they are described sufficiently, including the full set of available research data or analytical data. Therefore, methodical aspects and scientific reasons favor combining of or linking of a sample archival system and a research data repository. Such a combination was realized by using the Chemotion repository and the Molecule Archive, both located at Karlsruhe Institute of Technology (KIT). Chemotion repository is a repository for research data related to chemistry, particularly experimental chemistry. Scientists can either upload data directly or interoperably transfer it from an electronic lab notebook to the repository without losing information. The data are peer-reviewed in combination with automatic checks and can then be disclosed with persistent identifiers^21^. Especially for organic chemistry, the repository offers additional functions for direct viewing and analysis of the stored data by discipline-specific research software^22^ and thus enables an easy way of reusing the data. The repository follows an open access policy and is part of the strategy of NFDI4Chem in Germany.

The Molecule Archive is a facility of the KIT which enables the registration, validation, and collection of chemical substances. The substances are preserved for documentation and reuse purposes; therefore, strategies for sharing of the material and its provision have been developed. The use of the services of the Molecule Archive and the Chemotion repository is free of charge.

**Fig. 3:**
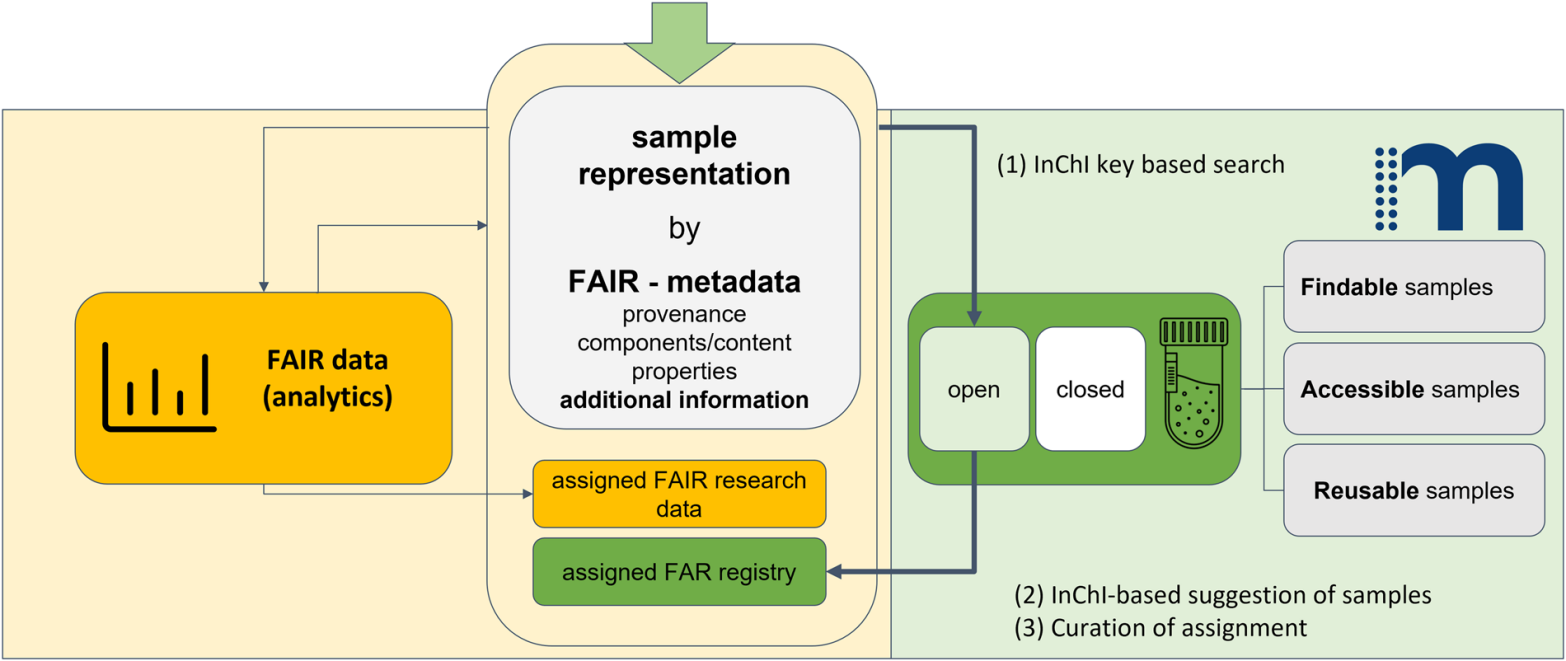
Technical implementation of the concept described in Fig. 2. The Chemotion repository is used as an infrastructure component to make research data and samples findable and accessible through one search entry, which is built on the sample representation. [ref Icons 3: Tube: *Digithrust from the noun project*, Data: *Popular from the noun project*].

In context of the concept described herein, we make use of the infrastructure afforded by Chemotion repository (as a research data repository) to publish data and metadata of samples as the primary data entity, including generation of associated DOIs, to obtain a virtual sample representation that makes physical samples findable. This virtual sample representation is matched with the physical samples in Molecule Archive by exchanging information between it and the repository via a defined protocol. This protocol checks if samples’ virtual representation in the Chemotion repository can be linked with registered physical samples in the Molecule Archive (Fig. 3, step (1)). The current request is based on the InChI key of a molecule, which is one of the most precise structural descriptors for chemical compounds. As a result, samples in the Molecule Archive, which have a sample representation in the Chemotion repository, are identified and information on their availability can be added to the information in the Chemotion repository. To this very general concept, a few saliencies have been added: Since not all samples provided to the Molecule Archive are intended to be publicly visible, the visibility of samples depends on the assignment of samples to an open sample collection in the Molecule Archive. Only samples within the open sample collection of the Molecule Archive are queried through the Chemotion repository (Fig. 3, query to “open” collection given in green, right panel) and then visible through the repository’s graphical user interface (GUI) (Fig. 3, step (2)). As the query is based on the InChI key of the molecule, the query may result in different suggested samples matching the InChI key. Therefore, linking of a sample in Chemotion repository to its physical counterpart is additionally curated by the Chemotion repository team (Fig. 3, step (3)).

#### Application and use of the infrastructure

**Fig. 4:**
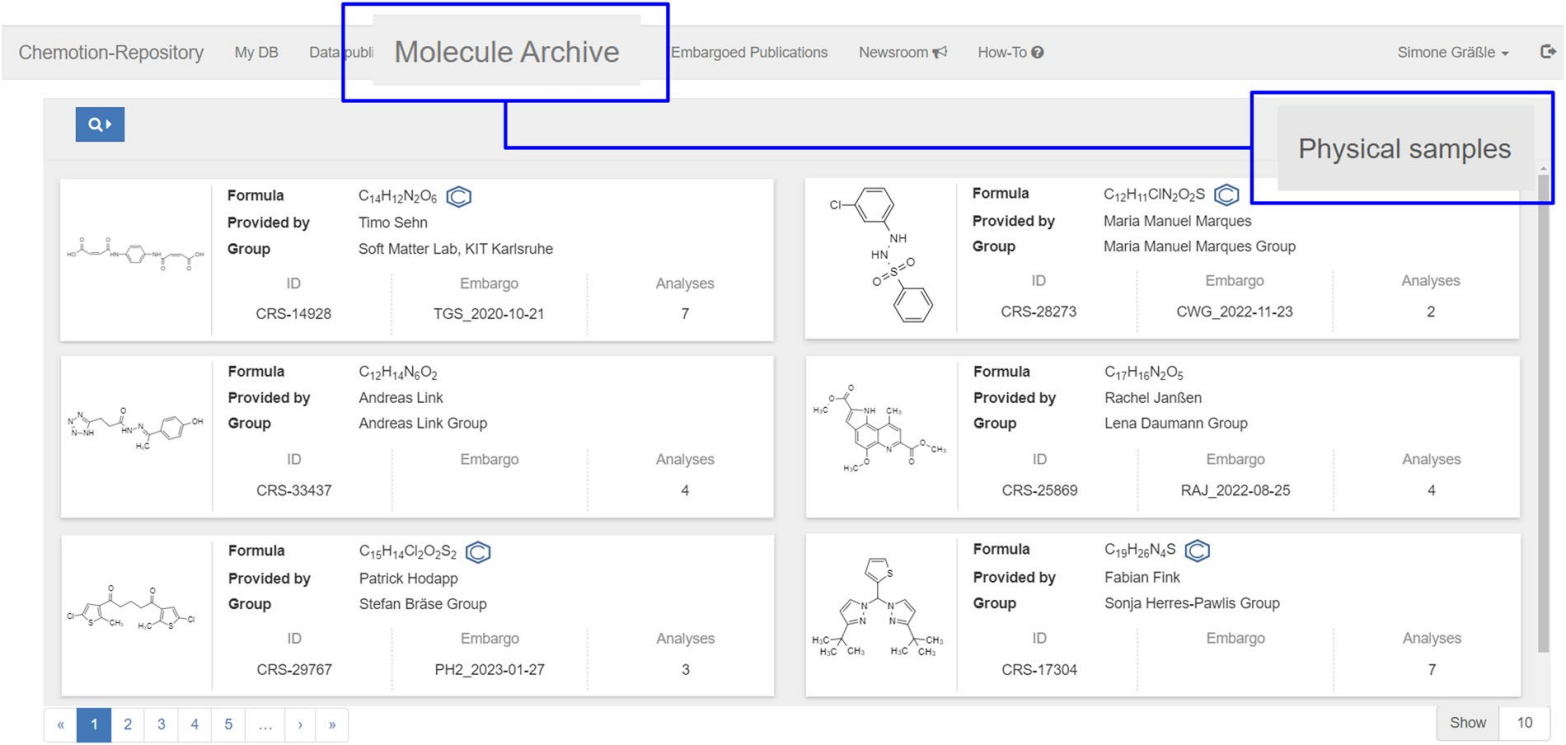
Overview of available material summarized in the table *physical samples* as given in the GUI of Chemotion repository in the section *Molecule Archive - physical samples*. The examples were collected and reorganized for this figure to reference different contributions (the entries were obtained in the repository Chemotion in a different order).

**Fig. 5:**
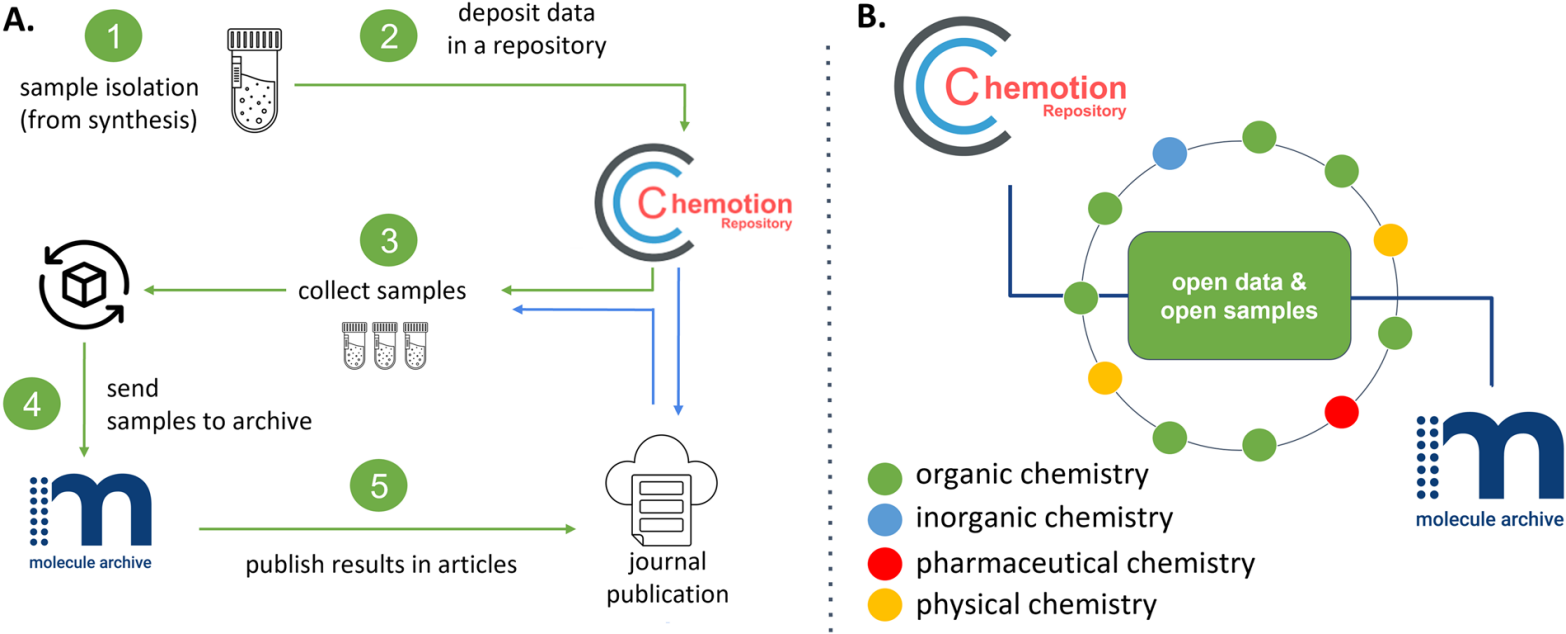
(**A**) Proposed workflow to gain FAIR-FAR samples along with the publication of scientific results and data. The workflow consists of five steps, from preparing samples and their characterization to depositing data and physical material, which can be done according to the suggested order (green) or other alternatives (one example given in blue). Following the green workflow offers the option to publish scientific results with reference to the research data and the reference to research materials. (**B**) The scientific network that contributed to the workflow and infrastructure design as described in this article with examples from different subdisciplines in chemistry and requirements from different groups and sites (details given in the SI, File 2). [ref Icons 4: parcel: *Adrien Coquet*, tube: *Digithrust from the noun project*, publication: *Vectors Lab*].

The infrastructure as described was established at KIT and is used by different scientists dealing with the synthesis of chemical compounds and, therefore, producing chemical samples. The established process was applied to more than 1400 chemical compounds, including examples from organic chemistry to inorganic compounds^23–26^, and metal-organic frameworks (MOFs)^27^. Each compound is a well-characterized product of a chemical reaction that was conducted for a specific project or research aim. Most of the compounds were published as part of a chemistry study before or after their submission to the Molecule Archive and the deposition of the corresponding data to the Chemotion repository. Scientists from different institutions tested the robustness of the infrastructure with respect to digital transfer and deposition of data, as well as physical transfer and deposition of samples (Fig. 4). They contributed to adapting of the infrastructure components to their scientific needs. The proposed process to obtain FAIR-FAR samples includes five steps: (1) Isolation of samples from a chemical reaction or a natural product isolation and their analytical characterization, (2) entering of the samples’ metadata and the deposition of the analytical data in a research data repository, (3) collection of samples and (4) shipping them to the location of the materials’ archive (in our example: Molecule Archive at KIT) including registration of the samples in the archive by the archive’s staff, and finally (5) publication of the results in scientific journals (Fig. 5A). The partner sites and research groups (circles) that helped establish the described FAIR-FAR samples model by providing the first use cases for open data and open materials in different subdisciplines in chemistry are represented schematically in Fig. 5B. The implemented process and the order of accompanying proposed steps is aligned with the demands of the research system in Germany, including new requirements to openly provide research data alongside scientific publications^28,29^. According to this requirement, the research data should be submitted to a repository before or at least in parallel with the publication of manuscripts in scientific journals in order to allow access to data during the review of publications. Therefore, step 2 needs to be finished before step 5 - even though data might be restricted to reviewers at this stage. The order of publication and materials’ provision can vary flexibly depending on the amount of material available and the intended publication strategy. Archiving the material before scientific results are published (green workflow in Fig. 5A) could strengthen the publication, as the materials’ archive can confirm the provision of the sample and provide information on the quality/purity of the samples. Further, it then becomes possible to refer to the compounds in the associated publication(s) – very similar to the referencing of data deposition in repositories. On the other hand, it may be preferable to deposit materials after publication of scientific results, i.e. once review is finished, especially when only a limited quantity of material is available (blue arrows in Fig. 5A). This has the advantage that materials are available at hand if reviewers need additional data from the authors.

The scientific network described in Fig. 5B helped fine-tune the proposed workflow of Fig. 5A and demonstrated that central availability of data and materials enables sharing of results with other scientists. We now discuss the impact of infrastructure and workflows described here on open and sustainable science in terms of findability, accessibility, interoperability and reusability of the materials metadata given in the sample representation as well as the findability, accessibility and reusability of the physical material itself.

### Findable sample metadata

The materials available within the Molecule Archive become manually and machine searchable through the samples’ metadata generated in the Chemotion repository. The repository’s GUI provides options for searching text and chemical structure, allowing users to find the available metadata based on the samples’ description which consists of provenance information, properties and the components given as molecular descriptors. Along with the samples’ virtual representation, the associated analytical research data, the physical location and IDs of the materials are findable. The sample representation is assigned a DOI, and its metadata includes this DOI. The metadata scheme (adopted from DataCite) also contains additional provenance information, physical descriptors characterizing the sample, and the information on the ID of the physical sample as registered in the Molecule Archive. Also included are the DOI to the chemical reaction data that generated the sample (if available) and the DOIs to associated analytical details. Metadata in the scheme are assigned to terminologies of established ontologies such as CHMO^30,31^, CHEMINF^32^, OBI^33^, and ChEBI^34^ wherever possible. In addition to being downloadable through the user interface of the repository, the metadata is also accessible via an Application Programming Interface (API) using a metadata harvesting protocol (OAI-PMH)^35^. Two examples of typical metadata schemas have been included with the Supplemental Information (SI, Fig S1 and Fig S2).

**Fig. 6:**
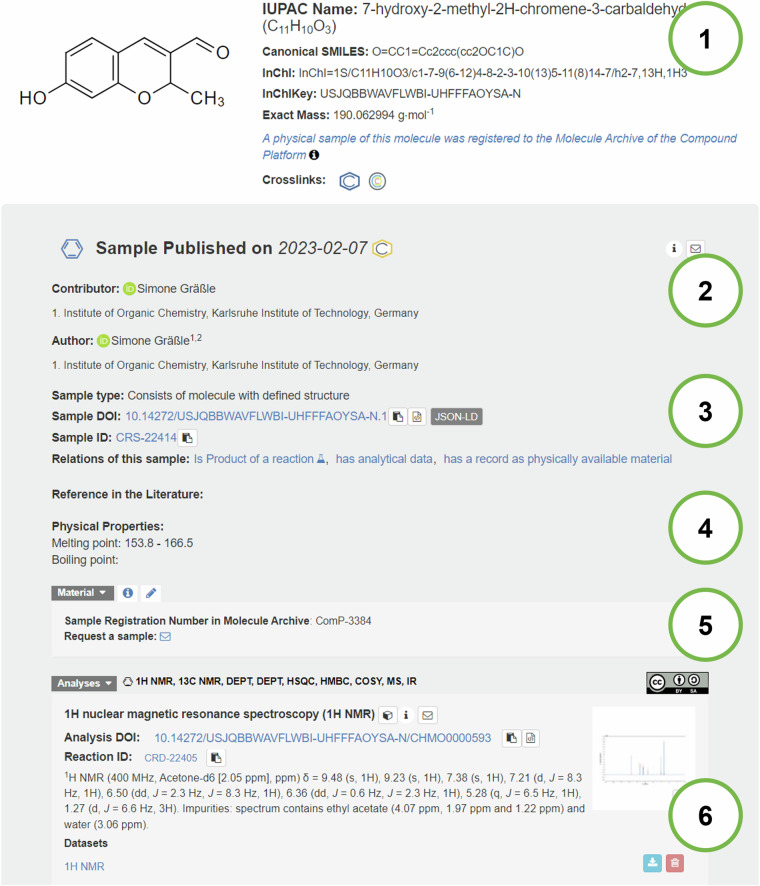
Explanation of the main parts that are used to describe a sample in the publication view of Chemotion repository: (1) Formal description of the sample’s virtual representation by information on the molecule which is part of the sample; (2) general publication metadata; (3) sample’s identifiers in Chemotion repository, (4) Selection of physical properties, (5) Access to the sample by contacting the team of the Molecule Archive and sample’s ID in the Molecule Archive; (6) Links to the analytical data that were gained with the sample. The Figure was created from a screenshot from the Chemotion repository and cut/changed to obtain an image that uses less space for a better readability of the article (see SI section 6 for the original screenshot as obtained).

The OAI-PMH protocol is used because of its broad usage and well-defined procedure. However, it also has some disadvantages such as the need to run and manage a dedicated OAI-PMH endpoint resulting in increased operational complexity. In addition, OAI-PMH is less scalable for very large datasets or frequent metadata updates as it relies on repeated harvesting. Therefore, in the future, accessibility to the metadata will be improved by including JSON-LD metadata schemas. The benefit of using JSON-LD with community-agreed schemas is that they are better at providing semantically rich domain-specific metadata and the linking of property values to defined terms from ontologies is more explicit. JSON-LD is also simpler to implement than OAI-PMH because it can be embedded directly in web pages and can be accessed via HTTP, eliminating the need for a specialized server or protocol making it faster and more scalable in the long run^36^. A first draft of such a JSON-LD implementation for samples was embedded in the samples’ representation in the Chemotion repository (Fig. 6, part 3; further information can be gained from SI section 5) and will be the subject of further discussion and improvement through the community of NFDI4Chem^37^.

### Accessible sample metadata

The Chemotion repository supports the OAI-PMH protocol, which is a widely used protocol for exposing metadata records in a standardized way that can then be harvested and aggregated by other systems. OAI-PMH provides access to metadata records at various levels, including individual and sets of records. For example, by using the “ListRecords” verb and applying filters such as the metadata prefix “oai_dc” and a date range users can obtain a list of complete records in Dublin Core format from the repository. Similarly, by using the “GetRecord” verb and applying filters such as the metadata prefix “oai_DataCite” and specifying an identifier like a sample’s DOI, users can retrieve a specific sample record, and therefore, access specific metadata from the Chemotion Repository in DataCite format. The protocol supports multiple metadata formats, such as Dublin Core and DataCite which allow easy interoperability of the Chemotion repository with other systems such as the NFDI4Chem search service^38^. Once the user finds the required sample information, all available metadata are directly accessible by their DOI identifier without further authentication and authorization processes. The metadata remains accessible, even when the materials are no longer available, and allow for a constant link to the analytical data.

### Interoperable sample metadata

Interoperable metadata are needed in particular to compare the materials with other available materials and to query additional databases. The sample metadata in the Chemotion repository include standardized molecule descriptors following domain-specific standards such as InChI, InChI keys and canonical SMILES strings wherever applicable. The terminologies used in the description of the samples are chosen from domain relevant ontologies and are assigned to the identifiers of the mentioned ontologies.

### Reusable sample metadata

The metadata for the sample in the Chemotion repository are described as comprehensively as possible, including description of the components characterizing the samples, their properties, and additional references to analytical data and synthetic origin of the sample (chemical reaction). In particular, the reference to analytical data and reactions allows reuse of the data since this information is needed for chemists to reproduce the experiments. A plurality of additional data that further describes the sample can be gained from the references to analytical data and the synthetic origin of the sample.

To gain FAIR-FAR samples, the FAIR metadata for samples is supplemented with measures to obtain findable, accessible and reusable materials/samples:

#### Findable materials

Labeling materials and cataloging of these labels in an accessible database are two essential steps to make materials findable. A clear and unique label or code (such as a barcode or a QR code) makes materials findable in a certain storage location by humans. As a second step, the material needs to be cataloged in a local database that keeps a certain minimum set of metadata about the material, including at least its label and the location where the material is stored. In our case, the registration is achieved by entering the samples’ metadata into the database of the Molecule Archive, making the samples findable by machines. As soon as the samples are physically deposited and registered in the Molecule Archive, the external findability of the available samples is further managed via the website of the Chemotion repository which allows the linkage of the unique label in the Molecule Archive with the metadata of the samples’ FAIR metadata which are visible globally.

### Accessible materials

Scientists interested in reusing the samples can place their request for sample directly through the repository’s interface (Fig. 6, details added to the SI, chapter 2). A contact form has been set up for each available sample to obtain the chemical compound in the form of a part of the sample. The query automatically transfers the identifier of the sample. A key difference in the notion of “accessible” for data and materials is the following: While access to data can be granted to all interested persons without disadvantage in each case, prioritization must be made with respect to access of materials. Since the amount of an available chemical compound archived per sample is usually very limited, there must be a consideration of the purposes for which the material should be released. For the Molecule Archive, the decision on whether to release the materials or not is made either based on a material transfer agreement (MTA^39^) of compound providers with the Molecule Archive (see chapter “reusability”) or is decided by the compound providers (and managed by the operators of the repository). The decision is sent to the interested reusers of the chemical compounds and if the material can be sent, the details and conditions for such reuse are clarified.

### Reusable materials

The reuse of samples available in the Molecule Archive is supported by quality assurance measures, framework agreements, consulting, and support in handling. For quality assurance, the registered samples are checked for purity and identity. The registration of the materials is not completed until the substances are physically available at the Molecule Archive and their identity and purity are checked. In contrast to sharing of data which can be managed by suitable licenses, the sharing of material needs material transfer agreements that clarify the role and rights of materials’ providers, materials’ reusers, and the Molecule Archive. The Molecule Archive supports the reuse of samples under a legal framework by providing a standardized material transfer agreement that was agreed on by KIT with different exemplarily chosen universities and published as a reference for further collaborators^39^. Further, the Molecule Archive organizes the communication between the participating scientists as needed. After clarification of all technical and legal issues, the provision of the material for subsequent use is initiated by preparing for sample transfer. The materials are then shipped according to common standards for chemical compounds.

## Discussion

Using examples from the subdisciplines of organic, pharmaceutical, inorganic, and experimental physical chemistry, a model was designed to provide data and materials of research results. The described approach is intended to be a possible first step towards more sustainable scientific work in chemistry, but currently, some challenges remain unsolved or are insolvable and impose a permanent limitation on the endeavor:

1. In principle, samples that do not tend to decompose under ambient conditions and are not volatile can be registered and recorded by the Molecular Archive. Currently, unstable metal-organic compounds in particular cannot be introduced as openly accessible and reusable samples. Storage under an inert gas atmosphere could expand the model’s applicability to include a wider range of substances; however, this is not done in the present setup.
2. So far, the method has only been used to provide data and materials for samples with a defined chemical structure. Therefore, mixtures or natural extracts have not been submitted as FAIR-FAR samples yet. This is, in principle, possible but the Chemotion repository is currently geared towards pure substances and will be adapted in the medium term.
3. While the registration and also the analysis of more complicated compound classes such as Metal-Organic-Frameworks (MOFs) is possible and the described infrastructure can be used to store and preserve MOF materials and data, the infrastructure is not well-suited for related samples such as SURMOFs (Surface-anchored MOFs). The infrastructure and concepts can still be used also for samples beyond their current scope - such as SURMOFs - but the physical archival and the evaluation of the gained material would need further solutions.
4. In some areas of chemistry, samples are generated that cannot be unambiguously described by any unique chemical structure. In such cases, the FAIR-FAR sample process described here can be used without restrictions, making the samples discoverable by DOIs. However, samples in these cases cannot be searched as efficiently as possible for samples with unique chemical structures because they would lack structural descriptors in the metadata.
  Other obvious hurdles for accessible and reusable samples in such cases arise from their limited availability, the resulting need for coordination to release the samples, and additional legal, technical, and security aspects.
5. While FAIR data can be reused almost indefinitely by granting a license and choosing the appropriate data infrastructure with regard to the number of reusers, various interests may have to be weighed against each other for the reuse of material - and the resources may be exhausted even if there is a need for further reuse. Currently, the Molecular Archive cannot provide a universal solution to this problem because the reuse scenarios are different, and decisions must be made on a case-by-case basis to achieve the highest benefit for the available substances. A well-organized distribution system must ensure that a certain amount of reference substance is kept, even if a high request for the compounds (samples) exists. This allows a residual amount to be available as analytical evidence independent of subsequent users of the substances. Still, the sample is linked to the synthesis protocol in Chemotion, as last aid in the case of depletion.
6. The provision of substances for reuse purposes requires time for preparation, at least at the time of the first provision of materials, due to the usually required MTA between the partners involved. The provision of samples therefore also depends on the processing time by the respective organizational units of the partners involved, if a legal basis for material reuse is to be created as it is done when licensing research data.

Despite the obvious challenges of providing materials, the combination of FAIR data and FAIR-FAR materials reveals enormous potential for more transparent and sustainable research. In particular, experimental disciplines in natural sciences may benefit from a concept introducing models for a systematic provision of samples. If samples are submitted before the publication of results, the benefits could be increased as the sample deposit can be directly linked to the publication. This strengthens the trust in the research work and allows direct linking of publication(s) to all its results: research data as well as the physical material.

Knowing that the implementation of the FAIR data principles is still far from completion, the additional push for FAIR-FAR samples might seem to be challenging. Nevertheless, the establishment of a standardized process for FAIR-FAR samples can be done very easily once the concept of FAIR data is adopted: the infrastructure supporting the access and reuse of chemical samples already exists, and the effort for a single scientist as the material’s producer is low if the initial agreements between the partner sites and the Molecule Archive are already in place. While the deposition of FAIR data currently lacks incentives for the providing scientists, the provision of materials offers special scientific advantages such as publications with materials’ reusers - this makes materials storage and provision an attractive aim that could foster the broad application of the FAIR-FAR concept. The provision of samples to the Molecule Archive has been the origin of many publications that were done in collaboration with compound providers and reusers – proving that the provisioning can result directly in more visibility and impact for the scientists sharing their research results^40–46^.

## Methods

### Software

The infrastructure described in this article is built with the use of open source software that was developed at KIT and has been described in previous articles. Both the Chemotion repository and the software behind the Molecule Archive were developed based on components of the source code of Chemotion ELN^47,48^. Further extensions of Chemotion ELN, including submission and reviewing workflow, provide the necessary functionality to operate the Chemotion research data repository^21,49^. The source code for the Chemotion repository can be obtained from GitHub^50^. For the operation of the Molecule Archive, Chemotion ELN was also used and adapted with a plugin^51^ to keep additional information on the sample information as provided by the owner and was extended with Foreign Data Wrappers (FDW)^52^ for smooth integration of data from the Chemotion repository and the Molecule Archive. An archived version of the source code of the Chemotion repository, as used for the work described in this article, can be obtained from Zenodo^53^.

### Submission of data to the Chemotion repository

The submission of data to the Chemotion repository enables the generation of the FAIR metadata of the samples’ virtual representation and the storage of FAIR research data. The data uploaded to the repository includes a request to the user to add information on the sample that was used for the measurement of the data. Usually this is the chemical structure of the compound assigned to the sample and additional information on the purity of the sample and other characteristics. This information is used by the software to automatically generate further sample metadata that can be used to identify the sample and to search for the sample. The combination of information entered by the user and system-generated information directly forms the virtual metadata representation of the sample and defines the digital object. With the information available about the sample, the submission of research data can be started. The data has to be prepared according to discipline-specific standards, described in detail in the online documentation of the Chemotion repository^54^. As soon as the virtual sample representation is visible (along with the data) after the publication of the submission, the correlation of research data and materials can be started by the team of the repository.

### Setting up a legal framework for sharing materials

Sharing of materials in a scientific environment can be done in two ways: either via a donation of the material from one scientific group to another one, or *via* the transfer of material under certain negotiated conditions. The FAIR-FAR samples concept supports both ways of sharing materials. Establishing the transfer of materials under an MTA costs more time and effort at the beginning of the sharing process – but enables the provision and reuse of compounds under clearly defined rules and is therefore the preferred way of sharing materials. Together with five exemplarily chosen partner sites, KIT created a standard MTA several years ago, which is now used as a routine process to introduce new partner sites to provide materials to the Molecule Archive^39^. The MTA outlines the rights and obligations among the material providers, the entity managing ComPlat and the reuser. It regulates the handling of compounds as well as data, and the publication of results in good scientific practice. Scientific credit is in particular important if the reusers of the materials gain scientific results with the provided compounds, and these results should be published together. The MTA emphasizes that reuse shall aim at publication and noncommercial purpose. A notification and non-disclosure retention period allows the preparation of publications and, exceptionally, the patenting of inventions that may involve the material or a related process. Where patentable inventions of a provider are inseparably linked to the scientific results of the reuser, the latter shall contractually perceive a contractual license option against conditions customary on the market. This compensates for the tax-payer funded prior-efforts, in accordance with state aid law. Altogether, more than three dozen research groups at universities and other noncommercial research entities are already partners of the Molecule Archive network, and scientists working at these sites can work under the existing MTA. Other scientists who work at institutions that do not have an agreement yet can request to start the MTA generation process with their institution.

### Submission of samples to the molecule archive

The submission of samples to the Molecule Archive works *via* a simple workflow: The Molecule Archive provides suitable standard vessels that are sent to the compound provider. The vessels are calibrated and carry a unique number for their later identification. A table sheet is provided to the users, which is required to register the sample in the database of the Molecule Archive. The sample providers need to give brief information on the chemical structure assigned to the materials in the form of the corresponding SMILES code, the code of the used vessel, the internal laboratory ID and properties such as the approximate purity of the material and the filled mass (an example is added to the SI, section 4). The filled vessels are then sent back to the Molecule Archive using the provided packaging material and the digital upload form is transmitted *via* email.

### Registration of samples in the molecule archive

The registration of the samples in the database of the Molecule Archive is done by the team of the Molecule Archive after the arrival of the material and works *via* upload of the table sheet to the database of the Molecule Archive. The Molecule Archive team checks the identity and purity of the compounds via LCMS (liquid chromatography coupled with mass spectrometry) and other techniques if required. If the data correspond to the provided structure of the sample-associated compound, the sample registration is finished, and the provider receives documentation about the submission and the results of the quality control. If the sample provider decides to make the sample openly accessible, the database entry for the sample is assigned to the collection of open samples within the database. The collection can then be accessed through the Chemotion repository and the sample is visible in the user interface of the repository along with the virtual sample representation (as depicted in Fig. 6 and the SI).

## Supplementary information

Supplementary Information

170 FAIR-FAR samples

## Data Availability

The Supplemental Information (SI, file 1) includes two examples as representative metadata schema of a virtual sample representation available in the Chemotion repository (chapter 1), and a detailed description of how the samples visible in the Chemotion repository website can be assessed for further reuse through the provided request form (chapter 2). Further, SI - part 1 covers further additional information on the processes of the Molecule Archive (chapter 3) including a template of a data upload form that is used to register samples in the Molecule Archive (chapter 4), an example for the currently implemented JSON-LD description (chapter 5), and the complete images of Fig. 6 in this article (chapter 6). The SI (file 2) contains an exemplarily collected and non-comprehensive list of partners and their contributions to the FAIR-AR samples concept. To give examples for the work described here, 170 examples out of ca. 1400 FAIR-FAR open samples (accessed on June, 5 2023, https://www.chemotion-repository.net/welcome) are cited in file 2 to allow direct access to some examples.

## Code availability

The code of the software that was adapted for this project is available as open source: Chemotion repository: 10.5281/zenodo.8028033; Molecule Archive functionality in Chemotion ELN code: https://gitlab.kit.edu/kit/complat/xvial.
