# Supplementary material for: Linking Research Data with Physically Preserved Research Materials in Chemistry
Source: Sci Data. 2025 Jan 22;12:130. doi: 10.1038/s41597-025-04404-2 (PMC11754846; doi:10.1038/s41597-025-04404-2)
Supplement: Supplementary file 1 — Supplementary Information [file 41597_2025_4404_MOESM1_ESM.pdf]

Supplemental Information

Linking Research Data with Physically Preserved Research Materials in Chemistry

Content

1. Description of selected design concepts 2
    1.1 FAIR, FAIR-FAR or even FAIR2FAR 2
    1.2 Publication requirements to obtain DOIs for samples 2
    1.3 Design of the infrastructure 3
2. Examples for metadata schema (Findability of Sample metadata) 5
3. Sample requests to the Molecule Archive (Accessibility of Samples/Materials) 7
4. Substance Submission to the Molecule Archive of the Compound Platform (ComPlat): Process Description to gain accessible and re-usable samples 8
    4.1 Material Transfer Agreement and sample transfer preparations 9
    4.2 Substance registration and quality check 9
    4.3 Reporting of substance quality and provision to the community 10
5. Sample submission sheet 12
6. Example for a JSON-LD implementation for the sample 12
7. Original Screenshot Figure 6 13

1. Description of selected design concepts

1.1 FAIR, FAIR-FAR or even $FAIR_2FAR$

The design of the infrastructure includes two types of metadata and a recommendation that deals with physical samples. Our concept is based on

- FAIR sample metadata
- FAIR research data (which can be assigned to sample metadata)
- FAR physical samples (see Fig S1).

In the case of chemistry, in particular if chemical structures are a fundamental part of the scientific work, the publication of FAIR research data somehow already includes the publication of metadata on the sample that was used to gain the research data. In the case of the chemotion repository, the publication of research data results in the availability of a sample representation which we describe here as FAIR sample metadata and the FAIR research data that are assigned to the samples' metadata. In our opinion, this concept could be described as $FAIR_2$ approach. However, in the manuscript, we further refer to FAIR as this is more common and avoids misunderstandings. Adding the concept of the FAR recommendation to this approach, results in the claim for $FAIR_2FAR$ or more common FAIR-FAR processes that include FAIR sample metadata, FAIR research data and FAR physical samples.

1.2 Publication requirements to obtain DOIs for samples

Our approach in principle distinguishes between the sample's metadata (Fig S1, grey panel) and the metadata for data obtained with the sample (Fig S1, organge panel). Both could be published independent of each other with DOI generation, allowing to provide metadata on the sample only, connect it to the FAR samples, and add further research data thereafter. In the current implementation however, we require a minimum set of research data to be published with the samples' metadata to ensure that the submitted sample has a high quality. In chemistry, a minimum set of data is available in all cases where a chemical sample was obtained and a certain identity or purity is claimed. We currently require such a proof of quality to ensure that the infrastructure can serve its purpose in the long run. Over time, we will evaluate whether the registration and publication of sample metadata without analytical data should be supported. Neither technical nor workflow requirements would prevent this change in the future.

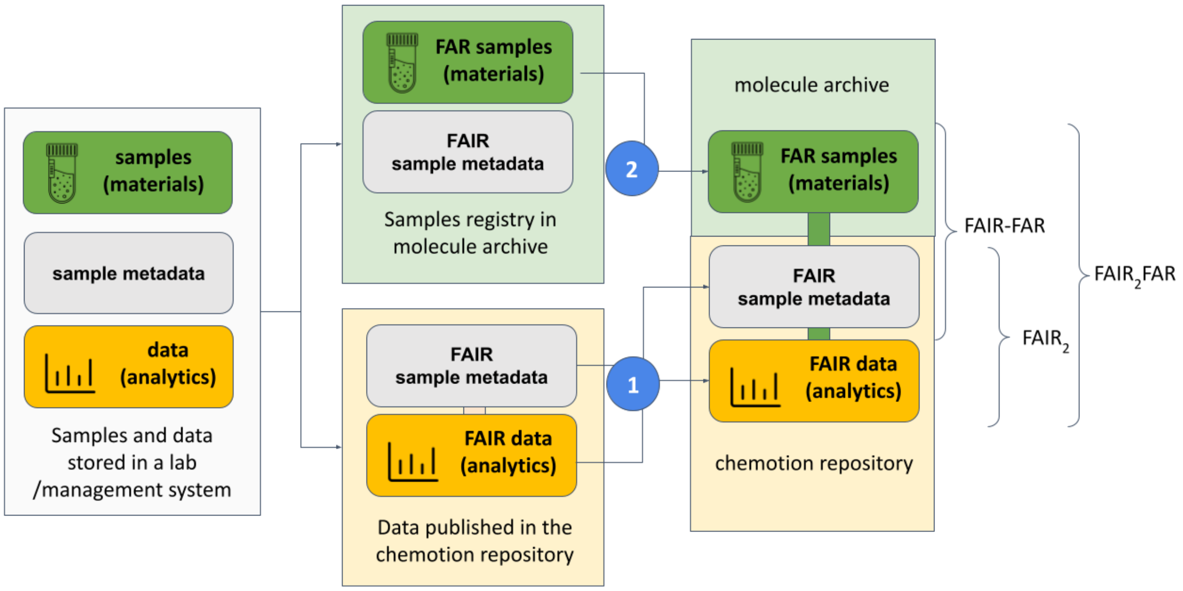

Fig S1. Schematic description of the processes supported by the concepts described in this manuscript. The final availability of FAIR sample metadata, FAIR data and FAR samples is gained by the linkage of the two infrastructures Chemotion repository and the Molecule Archive. The current process includes the publication of FAIR sample metadata with FAIR data in step 1, allowing then the assignment of FAR samples in step 2. The workflow is in principle independent of the provision of FAIR data, as the FAIR sample metadata does not depend on analytics. Nevertheless, the current process includes the submission of at least one dataset giving evidence of the samples' identity.

1.3 Design of the infrastructure

The design of the workflows and interactions of the infrastructures followed (a) general considerations of RDM initiatives, institutions and working groups that deal already with related challenges referring to the publication of research data and the handling of samples or materials. We considered (b) the specific requirements to such an infrastructure that arise due to the common practice of chemists in their scientific work, their publication strategies and specific challenges referring to the provision of data and materials. We further (c) designed the concept based on two already existing infrastructures, the chemotion repository and the molecule archive, both being established for many years and therefore taken as a basis for the design of the described workflows and principles.

Some selected details influencing the design with respect to point (a)

(1) For persistent identification of data and materials, the generation of PIDs is one of the most important aspects. The probably most often used service is DataCite, offering DOIs for research data but also samples.

-> according to our concept, DOIs are assigned to samples' representation and research data

(2) Therefore, suitable workflows and services need to be identified

-> in our concept, existing workflows of the chemotion repository are used as they were proven to be robust and accepted by the community

Some selected details influencing the design with respect to point (b)

(3) samples in chemistry are usually gained by either chemical reactions or the extraction of natural sources. Usually, they are a result of experiments and not considered

as naturally given reference samples - such as objects obtained directly from nature or cultural heritage.
-> not every sample obtained in chemistry is a sample that is worth to be considered for further reuse, and mechanisms to select the right ones need to be in place. In our case, we require proof of the quality and/or identity of the sample in the form of analytical data before samples are considered for the registration of a DOI.
(4) Chemical samples are obtained as a result of the daily work of the scientists - usually in a high frequency.
-> as a consequence of the need to check the quality of a sample before the generation of a DOI, far fewer samples can be prepared in a suitable manner by the sample providers to be registered through the repository used for the DOI generation. The DOI-service won't need to generate DOIs in a high throughput manner.
(5) The provision of materials in chemistry for re-use purposes usually requires some effort referring to the need for the purification of the chemical compounds and the effort to transfer them.
-> the required infrastructures need to be planned in a way that they can be extended in the long run, but there will be no scenario requiring a high troughput service in place for the Molecule archive.
(6) The handling of materials in an archive needs suitable systems that are capable of managing it properly and allowing the assignment of unique identifiers to samples also independent of the generation of DOIs which may be generated later in the work process.
-> our approach includes the provision of unique identifiers for samples in the Molecule Archive which can be used to connect the samples across different systems at different stages of their re-use.
(7) The publication of information on data and the sharing of materials follows the following principles:
a. samples are usually shared if there is at least one evidence (in form of a dataset) of the purity and the chemical identity of the sample (corresponds to the $FAIR_2FAR$ concept in Fig S1). Samples without are usually neither shared nor re-used (for certain valid reasons).
-> the desired concept of allowing the publication of samples with DOI in those cases where analytical evidence is provided in parallel is possible for the sample providers/producers as the data is usually obtained anyhow and can be provided alongside.
-> initiating the DOI generation for samples by the publication of at least a minimum of research data in the form of analytical information is helpful in providing services adapted to the special requirements of chemists to keep information confidential until the point where research data is published. The current system discloses samples' information only if the scientist as an owner of the material decides to release research data.
b. Data gained with chemical samples can be published with an information to which sample the data is assigned to, but in many cases, the samples are not available any more due to different reasons (corresponds to the $FAIR_2$ concept in Fig S1).
- > the publication of research data does not depend on the availability of the sample - but the information on the sample can be assigned as soon as it is made accessible.

Some selected details influencing the design with respect to point (c)
(9) The Chemotion Repository is already well established and part of a national strategy to preserve research data. The repository's workflows support the generation of DOIs that are assigned to the metadata of a sample and it allows for the publication of research data that belongs to samples (or processes - this second option is available but as not relevant here, it

won’t be discussed further). Research data is considered as data gained with samples but the data and sample metadata get separate DOIs. Also, different research data get different DOIs, allowing an easy differentiation of the data for citation purposes.
-> In our approach, the existing infrastructure Chemotion Repository is used for the publication of metadata on samples and will be further used for the publication of metadata on samples. Metadata on samples are required as a basis to link analytical research data (it’s current use case in the Chemotion Repository) and as a basis to reference the physical sample.
(10) The Molecule archive has been offering different services based on chemical samples to scientists for more than 10 years. It works based on the principle of data sharing where the Molecule Archive is the service that moderates the sharing and application of samples. The samples are registered to the archive and depending on the requirements, the sample’s metadata (and therewith the sample) are taken as confidential or non-confidential (information). Samples being available in the Molecule Archive are registered by providing a persistent identifier and they usually are available with analytical data - nevertheless, the archive has only access to some of the available information and the full set of analytical details is usually kept at the site of the sample owner.
-> The molecule archive should be used as a platform to find samples by machines such as the Chemotion repository - but only in those cases where the provider of the sample is ready to share this information. Also, the molecule archive should not manage publication processes, it should be used as a resource to provide information on the availability of samples based on selected metadata (such as the InChIKey at the moment).

2. Examples for metadata schema (Findability of Sample metadata)

Metadata schema for samples include a description part which consists of different sections. For samples that are described by a defined chemical entity, the description part in the metadata scheme contains (1) information on the type of sample [associated with a molecule or not], (2) a list of available structural descriptors for the assigned molecule if applicable, (3) further information describing if the sample is a dissolved chemical compound or a solvent free sample, (4) information on the unique ID of the sample in chemotion repository, (5) further physical descriptors of the sample, (6) the reference to the available analytical measurements describing the sample analytically, and (7) the list of ontologies from that terminologies were used to describe the metadata in a clear way.

Example 1: Sample DOI: https://dx.doi.org/10.14272/DGZQNPCSQDROIH-UHFFFAOYSA-N.1

```xml
<description xml:lang="en-US" descriptionType="Abstract">

  This is a physical chemical entity[CHEBI_24431] associated with a molecule[CHEBI_25367].
  The molecule[CHEBI_25367] can be described by the following structural desciptors[cheminf_000085]:
  InChI descriptor[cheminf_000113]: InChI=1S/C15H14Cl2S2/c1-8-12(6-14(16)18-8)10-4-3-5-11(10)13-7-15(17)19-9(13)2/h6-7H,3-5H2,1-2H3, and canonical SMILES descriptor
  [cheminf_000007]: Clc1sc(c(c1)C1=C(CCC1)c1cc(sc1C)Cl)C, and by the IUPAC name[cheminf_000107]: 5-chloro-3-[2-(5-chloro-2-methylthiophen-3-yl)cyclopenten-1-yl]
  -2-methylthiophene.

  The physical chemical entity[CHEBI_24431] has a component solvent[CHEBI_46787] which is described by the canonical SMILES descriptor[cheminf_000007]:

  The physical chemical entity[CHEBI_24431] has the following Sample ID as registered in the research data repository chemotion (www.chemotion-repository.net, https://doi.
  org/10.25504/FAIRsharing.iagXcR): CRS-29773

  The physical chemical entity[CHEBI_24431] can be described by the physical descriptors [CHEMINF_000025]:
  Melting point descriptor[CHEMINF_000256]:
  Boiling point descriptor[CHEMINF_000257]:
  Refractive index descriptor[CHEMINF_000253]:

  The physical chemical entity[CHEBI_24431] can be further described by the following assays[OBI:0000070][CHMO:0001133]:

    CHMO:0000593 | 1H nuclear magnetic resonance spectroscopy (1H NMR)

    CHMO:0000595 | 13C nuclear magnetic resonance spectroscopy (13C NMR)

    CHMO:0000480 | electron ionisation mass spectrometry (EI-MS)

    CHMO:0000498 | high-resolution mass spectrometry (HRMS)

  The physical chemical entity[CHEBI_24431] was deposited to the Molecule Archive of the Karlsruhe Insitute of Technology (KIT) with the following Sample ID: ComP-20041

  Used ontologies:
  CHEBI - Chemical Entities of Biological Interest
  CHEMINF - chemical information ontology (information entities about chemical entities)
  CHMO - Chemical Methods Ontology
  OBI - Ontology for Biomedical Investigations
</description>
```

```xml
<relatedIdentifiers>

    <relatedIdentifier relatedIdentifierType="DOI" relationType="HasPart">https://dx.doi.org/10.14272/DGZQNPCSQDROIH-UHFFFAOYSA-N/CHMO0000593</relatedIdentifier>

    <relatedIdentifier relatedIdentifierType="DOI" relationType="HasPart">https://dx.doi.org/10.14272/DGZQNPCSQDROIH-UHFFFAOYSA-N/CHMO0000498</relatedIdentifier>

    <relatedIdentifier relatedIdentifierType="DOI" relationType="HasPart">https://dx.doi.org/10.14272/DGZQNPCSQDROIH-UHFFFAOYSA-N/CHMO0000480</relatedIdentifier>

    <relatedIdentifier relatedIdentifierType="DOI" relationType="HasPart">https://dx.doi.org/10.14272/DGZQNPCSQDROIH-UHFFFAOYSA-N/CHMO0000595</relatedIdentifier>

    <relatedIdentifier relatedIdentifierType="DOI" relationType="IsPartOf">https://dx.doi.org/10.14272/reaction/SA-FUHFF-UHFFFADPSC-DGZQNPCSQD-UHFFFADPSC-NUHFF-NUHFF-NUHFF-ZZZ</relatedIdentifier>

</relatedIdentifiers>
```


Fig S2. Example 1 for an excerpt from a typical metadata scheme of a sample, representing the description part of the metadata, and representing the related identifiers part of the metadata.

Example 2: Sample DOI: https://dx.doi.org/10.14272/YRYNFVOSQUJGMT-UHFFFAOYSA-N.1


```xml
<description xml:lang="en-US" descriptionType="Abstract">

  This is a physical chemical entity[CHEBI_24431] associated with a molecule[CHEBI_25367].
  The molecule[CHEBI_25367] can be described by the following structural desciptors[cheminf_000085]:
  InChI descriptor[cheminf_000113]: InChI=1S/C15H14Cl2O2S2/c1-8-10(6-14(16)20-8)12(18)4-3-5-13(19)11-7-15(17)21-9(11)2/h6-7H,3-5H2,1-2H3, and canonical SMILES descriptor
  [cheminf_000007]: O=C(c1cc(sc1C)Cl)CCCC(=O)c1cc(sc1C)Cl, and by the IUPAC name[cheminf_000107]: 1,5-bis(5-chloro-2-methylthiophen-3-yl)pentane-1,5-dione.

  The physical chemical entity[CHEBI_24431] has a component solvent[CHEBI_46787] which is described by the canonical SMILES descriptor[cheminf_000007]:

  The physical chemical entity[CHEBI_24431] has the following Sample ID as registered in the research data repository chemotion (www.chemotion-repository.net, https://doi.
  org/10.25504/FAIRsharing.iagXcR): CRS-29767

  The physical chemical entity[CHEBI_24431] can be described by the physical descriptors [CHEMINF_000025]:
  Melting point descriptor[CHEMINF_000256]:
  Boiling point descriptor[CHEMINF_000257]:
  Refractive index descriptor[CHEMINF_000253]:

  The physical chemical entity[CHEBI_24431] can be further described by the following assays[OBI:0000070][CHMO:0001133]:

    CHMO:0000593 | 1H nuclear magnetic resonance spectroscopy (1H NMR)

    CHMO:0000595 | 13C nuclear magnetic resonance spectroscopy (13C NMR)

    CHMO:0000480 | electron ionisation mass spectrometry (EI-MS)

  The physical chemical entity[CHEBI_24431] was deposited to the Molecule Archive of the Karlsruhe Insitute of Technology (KIT) with the following Sample ID: ComP-20042

  Used ontologies:
  CHEBI - Chemical Entities of Biological Interest
  CHEMINF - chemical information ontology (information entities about chemical entities)
  CHMO - Chemical Methods Ontology
  OBI - Ontology for Biomedical Investigations
</description>
<relatedIdentifiers>

  <relatedIdentifier relatedIdentifierType="DOI" relationType="HasPart">https://dx.doi.org/10.14272/YRYNFVOSQUJGMT-UHFFFAOYSA-N/CHMO0000593</relatedIdentifier>

  <relatedIdentifier relatedIdentifierType="DOI" relationType="HasPart">https://dx.doi.org/10.14272/YRYNFVOSQUJGMT-UHFFFAOYSA-N/CHMO0000480</relatedIdentifier>

  <relatedIdentifier relatedIdentifierType="DOI" relationType="HasPart">https://dx.doi.org/10.14272/YRYNFVOSQUJGMT-UHFFFAOYSA-N/CHMO0000595</relatedIdentifier>

  <relatedIdentifier relatedIdentifierType="DOI" relationType="IsPartOf">https://dx.doi.org/10.14272/reaction/SA-FUHFF-UHFFFADPSC-YRYNFVOSQU-UHFFFADPSC-NUHFF-NUHFF-NUHFF-ZZZ</relatedIdentifier>

</relatedIdentifiers>
```

Fig S3. Example 2 for an excerpt from a typical metadata scheme of a sample, representing the description part of the metadata, and representing the related identifiers part of the metadata.

3. Sample requests to the Molecule Archive (Accessibility of Samples/Materials)

Scientists who are interested in the re-use of the samples can search within the available compounds on the Chemotion repository page and can place their sample request directly through the repository's interface.

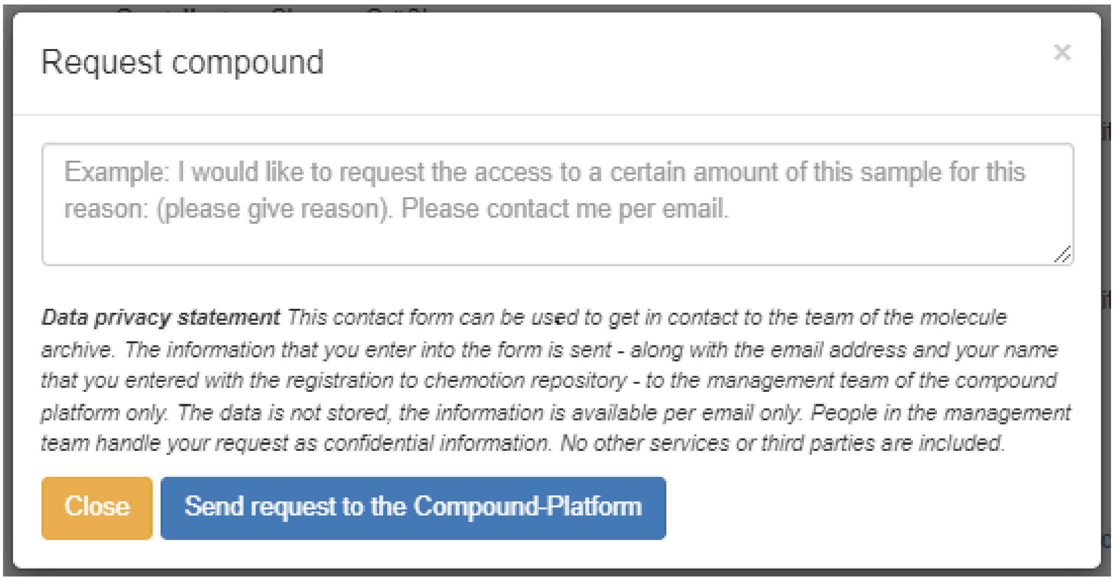

Fig S4. The request form available in Chemotion repository - enabling access to the samples stored in the Molecule Archive.

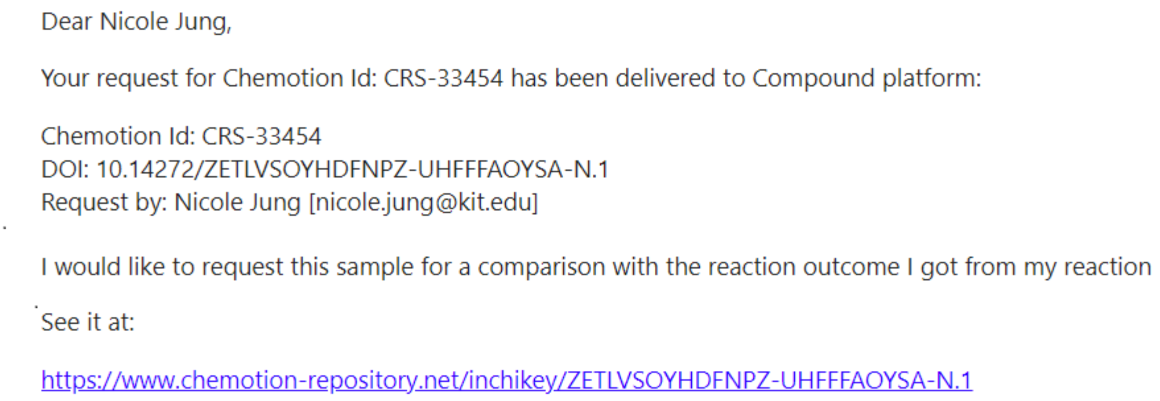

Dear Nicole Jung,

Your request for Chemotion Id: CRS-33454 has been delivered to Compound platform:

Chemotion Id: CRS-33454
DOI: 10.14272/ZETLVSOYHDFNPZ-UHFFFAOYSA-N.1
Request by: Nicole Jung [nicole.jung@kit.edu]

I would like to request this sample for a comparison with the reaction outcome I got from my reaction

See it at:

https://www.chemotion-repository.net/inchikey/ZETLVSOYHDFNPZ-UHFFFAOYSA-N.1

Fig S5. The request to the team of the Molecule Archive is confirmed by a notification sent per email to the requesting person.

Dear Compound team

Here is a request for Compound X-Vial: X12951 from Chemotion User [Nicole Jung]:

Chemotion Id: CRS-33454
DOI: 10.14272/ZETLVSOYHDFNPZ-UHFFFAOYSA-N.1
Compound X-Vial: X12951
Request by: Nicole Jung [nicole.jung@kit.edu]

I would like to request this sample for a comparison with the reaction outcome I got from my reaction

See it at:

https://www.chemotion-repository.net/inchikey/ZETLVSOYHDFNPZ-UHFFFAOYSA-N.1

Fig S6. The request is forwarded to the compound platform team.

4. Substance Submission to the Molecule Archive of the Compound Platform (ComPlat): Process Description to gain accessible and re-usable samples

The process of substance submission to the Molecule Archive consists roughly of three phases: 1) Transfer of samples to the Molecule Archive, 2) Quality check and substance registration, and 3) Reporting of substance quality and provision to the community.

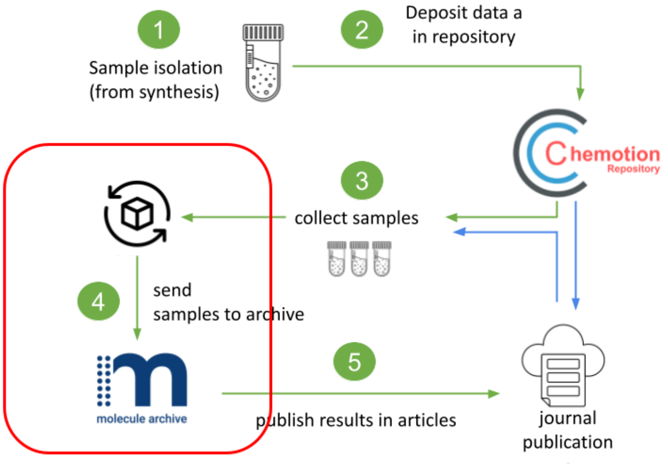

Fig S7. The section 3 of the supplemental information describes the process of sending samples from the provider (scientist) to the team of the Molecule Archive in detail.

4.1 Material Transfer Agreement and sample transfer preparations

Scientists that want to provide compounds to the Molecule Archive are supported by a legal framework that describes the rules and details of a provision of compounds and their re-use. The legal framework is generated in the form of a Material Transfer Agreement (MTA) that is provided by KIT and signed by both participating parties, KIT and the institution of the compound provider (https://dx.doi.org/10.35097/1022). It regulates the ownership of intellectual property, the handling of obtained or transmitted data and the publication of results in cases where other scientists re-use materials and want to publish the results that are gained. The contract template has been processed and agreed by over a dozen universities and research entities in the past, confirming the compatibility with the expectations and requirements of compound providers of different universities and the Molecule Archive.[1]
In parallel to the review of the MTA, the team of the Molecule Archive sends standardized vessels (X-vials) to the chemists that want to preserve their compounds. The vessels are previously balanced and are labelled with a specific unique number (X-number) for the later assignment of the compounds to the samples. Furthermore, a digital upload sheet is provided which will be used for the later transfer of information into the Molecule Archive database.

After the MTA is signed by both parties, the vessels with samples included are sent to the Molecule Archive and relevant sample information is added into the digital upload sheet. This includes the corresponding SMILES code of the substance, the label of the vessel used, the original laboratory internal ID (lab code), the approximate purity of the material, and the provided mass.

4.2 Substance registration and quality check

The second phase involves the digital upload of the chemical structures into the Molecule Archive database using the upload sheet. The chemical structures of the provided substances are uploaded to the database together with the information given in the digital upload sheet, however not yet assigned to the Molecule Archive. With the arrival of the compounds, the identity and purity of the substances are checked through a routine consisting of thin-layer chromatography and LCMS analysis. A substance purity of at least 90% is required for full acceptance and a registration to the archive's compound sharing program. This is usually determined during LCMS analysis, using UV-Vis detection at 230 nm. If the results from this measurement are inconclusive, other methods such as GCMS and NMR spectroscopy may be applied. The substance used for NMR spectroscopy is recovered. Compounds that pass the quality check are finally added to the

---

[1] Karlsruhe Institute of Technology, legal affairs unit. Agreement on the transfer of materials via the Compound Platform (ComPlat). Preprint at https://doi.org/10.35097/1022 (2023).

Molecule Archive and are stored as solid material as well as 10 mM DMSO stock solution at -20 °C to be ready to be re-used by other scientists. Compounds that do not pass the quality check are not preserved and provided automatically for further re-use. Depending on the amount of substance provided, The Molecule Archive team provides further purification of contaminated substances using preparative HPLC after consultation of the provider. Substances with less than 60% purity or when provided in insufficient quantity are returned to the chemist or discarded on demand.

4.3 Reporting of substance quality and provision to the community

Finally, the Molecule Archive reports the substance quality back to the providing chemist. This report contains an overview of the performed analyses, its results and a summary of accepted and rejected substances. Data that has been obtained during quality assessment are made available to the provider through deposition in the online data storage service of Baden-Württemberg (bwSync&Share). Accepted substances are accessible to the research community, but may also be withheld upon request. In general, there are two options that can be supported: (1) The provider of the compounds does not want to share the material openly, which means that the compound structures are technically part of the “closed” collections of the Molecule Archive (see Fig. 3, manuscript) and information on the availability of the material is not visible in the Chemotion repository. In those cases, further collaboration partners for distinct re-use cases are managed by the team of the Molecule Archive. (2) The providers of compounds agreed to assign the samples to the “open” collection of the Molecule Archive. This induces the option to be findable by the Chemotion repository and therefore visible *via* the Chemotion repository web interface (see Figures 5 and 6 in the manuscript).

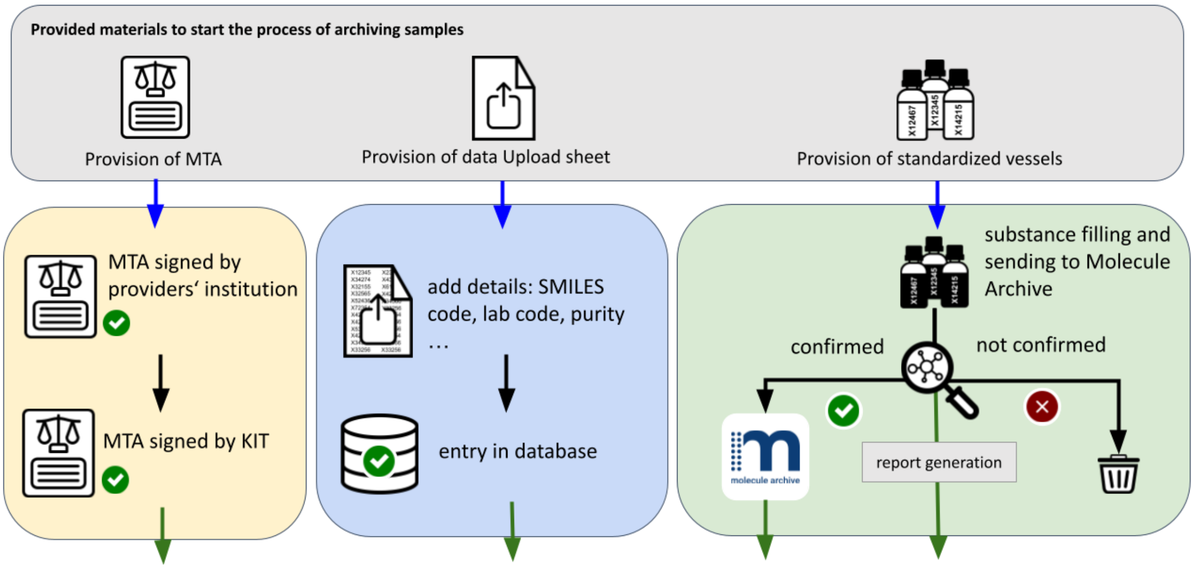

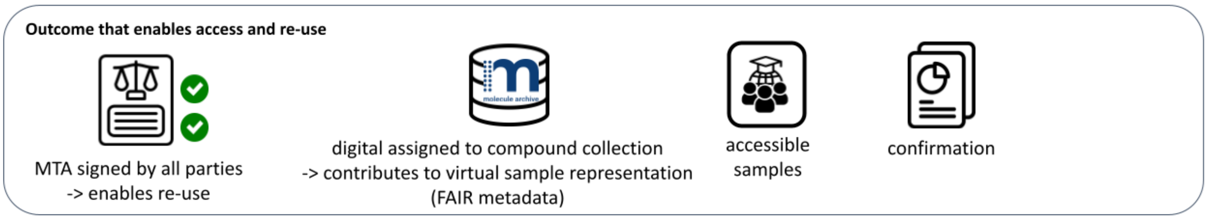

Fig S8. Schematic representation of the substance submission process to the Molecule Archive of the Compound Platform. Initially, the Compound Platform provides the respective chemist with calibrated and numbered vessels (X-Vials) for filling, as well as two documents, including the Compound Platform's Material Transfer Agreement and a digital upload sheet for substance-specific metadata. Each represents one starting point for three different working levels of the submission process, namely the legal level (yellow panel), the digital level (blue panel), and the practical level (green panel). The legal level (yellow panel) aims to conclude a Material Transfer Agreement as the basis for any exchange of materials and data between the chemist, the Compound Platform, and the scientific community. It is reviewed and signed by the provider's institution legal department and then forwarded to the KIT legal department for countersignature. On the practical level (green panel), the providing chemist fills the received X-Vials with substances and sends them back to the Compound Platform. There, the substances are routinely checked for identity and purity. The results of this analysis are reported back to the provider. If the substances meet the required purity standards, they are added to the Molecule Archive and made available to the scientific community for subsequent use, if desired. If the purity is insufficient, the substances are either sent back or disposed of in consultation with the provider. The digital level (blue) involves the acquisition and processing of relevant substance-specific metadata. The provided digital upload sheet is filled out by the chemist with information data that allow for the unique assignment of the physical samples to the digital entries in the Molecule Archive's database. This includes SMILES codes, corresponding X-numbers, original sample designation, and approximate purity of the submitted substances. Based on the information provided in the upload sheet, entries for the provided substances are then created in the database. For pure substances, these entries are finally assigned to the digital collection of the Molecule Archive and linked to the FAIR data publications in the Chemotion Repository.

References of Icons in **Fig S8** from the Noun Project:

“Approved” by Milinda Courey from https://thenounproject.com/icon/approved-1827000/
“Community” by Anggara Putra from https://thenounproject.com/icon/community-5742881/
“Failed” by karacis studio from https://thenounproject.com/icon/failed-3977955/
“Essential Oil” by Tyler Gobberdiel from https://thenounproject.com/icon/essential-oil-3114031/
“Legal document” by hazicon from https://thenounproject.com/icon/legal-document-4389665/
“Document” by Bryn Tylor from https://thenounproject.com/icon/document-101385/
“Upload” by Rahmat Sigit Prasetyo from https://thenounproject.com/icon/upload-1049546/
“Database” by ARISO from https://thenounproject.com/icon/database-4989223/
“Magnifier” by Riyan Resdian from https://thenounproject.com/icon/magnifier-5725569/
“Molecule” by very poernomo from https://thenounproject.com/icon/molecule-3782385/
“Report” by syarif from https://thenounproject.com/icon/report-5744730/
“Trash” by popcornarts from https://thenounproject.com/icon/trash-5744508/
“World” by anggun from https://thenounproject.com/icon/world-1744039/

5. Sample submission sheet

The sample providers need to give brief information on the chemical structure assigned to the materials in form of the corresponding SMILES code, the code of the used vessel, the internal laboratory ID and properties such as the approximate purity of the material and the filled mass. This information is given in a standardized way by using the upload form, which is cited as follows. The form is generated as part of step 3.1 (see previous chapter) and used/uploaded as part of step 3.2 (see previous chapter).

	A	B	C	D	E	F	G	H
1	(structure)[1]	smiles	source sample ID	X vial number	amount (mg)	Purity (%)	Comments [2]	
2		COc1ccc(cc1)c1ccccn1	SG-V3844 (17-23)	X22227				
3		Clc1ncnc(c1C)c1cccc(n1)c1ccccn1	SG-V3837 (14-17)	X22226				
4								
5								
6								

6. Example for a JSON-LD implementation for the sample


```
@context : https://schema.org
@type : ChemicalSubstance
@id : https://doi.org/10.5072/LBLPBDUCKUHMIA-UHFFFAOYSA-N.1
identifier : CRS-33248
url : https://www.chemotion-repository.net/inchikey/LBLPBDUCKUHMIA-UHFFFAOYSA-N.1
name : 8-(2,2-dichloroacetyl)-5-hydroxy-2,7-dimethylnaphthalene-1,4-dione
alternateName : InChI=1S/C14H10Cl2O4/c1-5-3-7(17)10-8(18)4-6(2)12(19)11(10)9(5)13(20)14(15)16/h3-4,14,17H,1-2H3
image : https://www.chemotion-repository.net/images/samples/4cf5595213d2ee78203da5d34e38f8d7293257db5d163d517cbd867a04ea11babca0494e2c029854baec2748cc31280a3f1ca62416390f5c985b98a49f30a5a3.svg
description : value
hasBioChemEntityPart : {
  @type : MolecularEntity
  smiles : ClC(C(=O)c1c(C)cc(c2c1C(=O)C(=CC2=O)C)O)Cl
  inChIKey : LBLPBDUCKUHMIA-UHFFFAOYSA-N
  inChI : InChI=1S/C14H10Cl2O4/c1-5-3-7(17)10-8(18)4-6(2)12(19)11(10)9(5)13(20)14(15)16/h3-4,14,17H,1-2H3
  name : 8-(2,2-dichloroacetyl)-5-hydroxy-2,7-dimethylnaphthalene-1,4-dione
  molecularFormula : C14H10Cl2O4
  molecularWeight : {
    @type : QuantitativeValue
    value : 313.1327999999999
    unitCode : g/mol
  }
  iupacName : 8-(2,2-dichloroacetyl)-5-hydroxy-2,7-dimethylnaphthalene-1,4-dione
```


DOI: https://dx.doi.org/10.14272/LBLPBDUCKUHMIA-UHFFFAOYSA-N.1
JSON-LD: download

7. Original Screenshot Figure 6

The following parts represent the complete submission of the sample that is shown in Figure 6 of the main manuscript. The submission can be retrieved under this DOI-link: https://dx.doi.org/10.14272/USJQBBWAVFLWBI-UHFFFAOYSA-N.1

The information was gained in different screenshots that were collected and added to one description.

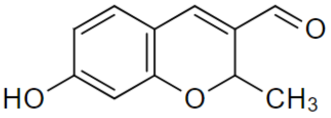

IUPAC Name: 7-hydroxy-2-methyl-2H-chromene-3-carbaldehyde ($C_{11}H_{10}O_3$)

Canonical SMILES: O=CC1=Cc2ccc(cc2OC1C)O

InChI: InChI=1S/C11H10O3/c1-7-9(6-12)4-8-2-3-10(13)5-11(8)14-7/h2-7,13H,1H3

InChIKey: USJQBBWAVFLWBI-UHFFFAOYSA-N

Exact Mass: 190.062994 $g \cdot mol^{-1}$

A physical sample of this molecule was registered to the Molecule Archive of the Compound Platform

Crosslinks:

Sample Published on *2023-02-07*

Contributor: Simone Gräßle

1. Institute of Organic Chemistry, Karlsruhe Institute of Technology, Germany
2. Institute of Biological and Chemical Systems - Functional Molecular Systems, Karlsruhe Institute of Technology, Germany

Author: Simone Gräßle[1,2]

1. Institute of Organic Chemistry, Karlsruhe Institute of Technology, Germany
2. Institute of Biological and Chemical Systems - Functional Molecular Systems, Karlsruhe Institute of Technology, Germany

Sample type: Consists of molecule with defined structure
Sample DOI: 10.14272/USJQBBWAVFLWBI-UHFFFAOYSA-N.1 JSON-LD
Sample ID: CRS-22414
Relations of this sample: Is Product of a reaction, has analytical data, has a record as physically available material

Reference in the Literature:

Physical Properties:
Melting point: 153.8 - 166.5
Boiling point:

Material

Sample Registration Number in Molecule Archive: ComP-3384
Request a sample:

Analyses 1H NMR, 13C NMR, DEPT, DEPT, HSQC, HMBC, COSY, MS, IR

1H nuclear magnetic resonance spectroscopy (1H NMR)

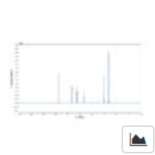

Analysis DOI: 10.14272/USJQBBWAVFLWBI-UHFFFAOYSA-N/CHMO0000593
JSON-LD
Reaction ID: CRD-22405

^{1}H NMR (400 MHz, Acetone-d6 [2.05 ppm], ppm) δ = 9.48 (s, 1H), 9.23 (s, 1H), 7.38 (s, 1H), 7.21 (d, J = 8.3 Hz, 1H), 6.50 (dd, J = 2.3 Hz, J = 8.3 Hz, 1H), 6.36 (dd, J = 0.6 Hz, J = 2.3 Hz, 1H), 5.28 (q, J = 6.5 Hz, 1H), 1.27 (d, J = 6.6 Hz, 3H). Impurities: spectrum contains ethyl acetate (4.07 ppm, 1.97 ppm and 1.22 ppm) and water (3.06 ppm).

Datasets

1H NMR

13C nuclear magnetic resonance spectroscopy (13C NMR)

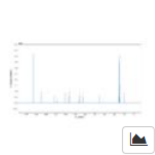

Analysis DOI: 10.14272/USJQBBWAVFLWBI-UHFFFAOYSA-N/CHMO0000595
JSON-LD
Reaction ID: CRD-22406

^{13}C NMR (100 MHz, Acetone-d6 [29.9 ppm], ppm) δ = 190.3 (CH), 163.4 (C_q), 157.1 (C_q), 141.1 (CH), 134.0 (C_q), 131.9 (CH), 113.6 (C_q), 110.6 (C_q), 104.5 (CH), 70.6 (CH), 20.2 (CH_3).

Datasets

13C NMR

distortionless enhancement with polarization transfer (DEPT)

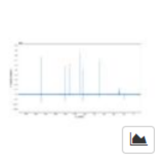

Analysis DOI: 10.14272/USJQBBWAVFLWBI-UHFFFAOYSA-N/CHMO0000596
JSON-LD
Reaction ID: CRD-22407

Datasets

DEPT90

distortionless enhancement with polarization transfer (DEPT)

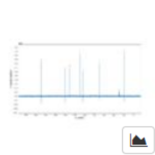

Analysis DOI: 10.14272/USJQBBWAVFLWBI-UHFFFAOYSA-N/CHMO0000596.1
JSON-LD
Reaction ID: CRD-22408

Datasets

DEPT135

heteronuclear single quantum coherence (HSQC)

Analysis DOI: 10.14272/USJQBBWAVFLWBI-UHFFFAOYSA-N/CHMO0000604

JSON-LD

Reaction ID: CRD-22409

Preview
not available

Datasets

HSQC

heteronuclear multiple bond coherence (HMBC)

Analysis DOI: 10.14272/USJQBBWAVFLWBI-UHFFFAOYSA-N/CHMO0000601

JSON-LD

Reaction ID: CRD-22410

Preview
not available

Datasets

HMBC

correlation spectroscopy (COSY)

Analysis DOI: 10.14272/USJQBBWAVFLWBI-UHFFFAOYSA-N/CHMO0000599

JSON-LD

Reaction ID: CRD-22411

Preview
not available

Datasets

COSY

mass spectrometry (MS)

Analysis DOI: 10.14272/USJQBBWAVFLWBI-UHFFFAOYSA-N/CHMO0000470

JSON-LD

Reaction ID: CRD-22412

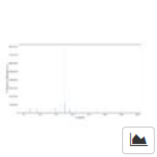

MS (EI, 70 eV, 80 °C), m/z (%): 190 (39) $[M]^+$, 175 (100), 161 (17).
HRMS–EI ($C_{11}H_{10}O_3$) *(m/z)*: $[M]^+$ Calcd 190.0624; Found 190.0625.

Datasets

Mass

HRMS

infrared absorption spectroscopy (IR)

Analysis DOI: 10.14272/USJQBBWAVFLWBI-UHFFFAOYSA-N/CHMO0000630

JSON-LD

Reaction ID: CRD-22413

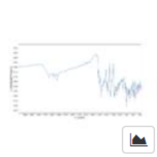

IR (ATR, ṽ) = 3097 (m), 2978 (m), 2925 (m), 2849 (m), 2839 (m), 1638 (w), 1602 (s), 1548 (vs), 1507 (s), 1465 (s), 1441 (m), 1392 (s), 1378 (s), 1361 (vs), 1279 (vs), 1231 (s), 1164 (vs), 1152 (vs), 1116 (s), 1096 (vs), 1075 (vs), 1035 (vs), 1004 (s), 975 (s), 943 (s), 909 (vs), 850 (s), 833 (s), 823 (s), 803 (vs), 768 (vs), 732 (vs), 722 (vs), 704 (vs), 642 (vs), 599 (vs), 585 (vs), 544 (vs), 511 (vs), 473 (vs), 456 (vs), 426 (s), 391 (vs), 380 (s) cm^{-1}.

Datasets

IR
