# Supplementary material for: Linking Research Data with Physically Preserved Research Materials in Chemistry
Source: Sci Data. 2025 Jan 22;12:130. doi: 10.1038/s41597-025-04404-2 (PMC11754846; doi:10.1038/s41597-025-04404-2)
Supplement: Supplementary file 2 — 170 FAIR-FAR samples [file 41597_2025_4404_MOESM2_ESM.pdf]

Datas	Location	author-group	repo sample ID	X-Nummer	molecule archive ID	DOI Repo
1	Lisboa	Maria Manuel Marques	https://www.chemotion-re	X20401	ComP-18270	https://dx.doi.org/10.14272/DUAQQPBEBZYIHR-UHFFFAOYSA-N.1
1	Lisboa	Maria Manuel Marques	https://www.chemotion-re	X20414	ComP-18257	https://dx.doi.org/10.14272/NQGYJZMMGBFFFC-UHFFFAOYSA-N.1
1	Lisboa	Maria Manuel Marques	https://www.chemotion-re	X20411	ComP-18260	https://dx.doi.org/10.14272/DGBYZIRNJBIXEI-UHFFFAOYSA-N.1
1	Lisboa	Maria Manuel Marques	https://www.chemotion-re	X20403	ComP-18268	https://dx.doi.org/10.14272/MVFSSJKMQUDTLZ-UHFFFAOYSA-N.1
1	Lisboa	Maria Manuel Marques	https://www.chemotion-re	X20415	ComP-18256	https://dx.doi.org/10.14272/JJOSXMIVEFQUSL-UHFFFAOYSA-N.1
1	Lisboa	Maria Manuel Marques	https://www.chemotion-re	X20409	ComP-18262	https://dx.doi.org/10.14272/SZZHGCPDOORRHI-UHFFFAOYSA-N.1
1	Lisboa	Maria Manuel Marques	https://www.chemotion-re	X20416	ComP-18255	https://dx.doi.org/10.14272/WKKLJJGGWZHEKG-UHFFFAOYSA-N.1
1	Lisboa	Maria Manuel Marques	https://www.chemotion-re	X20402	ComP-18269	https://dx.doi.org/10.14272/CGJMIHZEFDRIGG-UHFFFAOYSA-N.1
2	KIT	Frank Biedermann/Changming Hu	https://www.chemotion-re	X17305	ComP-15748	https://dx.doi.org/10.14272/RJLCCSUQZUOZTJ-UHFFFAOYSA-M.1
2	KIT	Frank Biedermann/Changming Hu	https://www.chemotion-re	X17309	ComP-15744	https://dx.doi.org/10.14272/ULNWEZYJTNMIQQ-UHFFFAOYSA-M.1
2	KIT	Frank Biedermann/Changming Hu	https://www.chemotion-re	X17306	ComP-15747	https://dx.doi.org/10.14272/LACSKPODWQXGSB-UHFFFAOYSA-N.1
2	KIT	Frank Biedermann/Changming Hu	https://www.chemotion-re	X17303	ComP-15750	https://dx.doi.org/10.14272/GYFSYEVKFOOLFZ-UHFFFAOYSA-N.1
2	KIT	Frank Biedermann/Changming Hu	https://www.chemotion-re	X17308	ComP-15745	https://dx.doi.org/10.14272/JROBPRZYLGNZEB-UHFFFAOYSA-M.1
2	KIT	Frank Biedermann/Changming Hu	https://www.chemotion-re	X17304	ComP-15749	https://dx.doi.org/10.14272/BTUNLJUIACBNND-UHFFFAOYSA-M.1
2	KIT	Frank Biedermann/Changming Hu	https://www.chemotion-re	X17307	ComP-15746	https://dx.doi.org/10.14272/ZGCIPCWPNLEHCL-UHFFFAOYSA-M.1
3	KIT	Patrick Hodapp	https://www.chemotion-re	X22561	ComP-20042	https://dx.doi.org/10.14272/YRYNFVOSQUJGMT-UHFFFAOYSA-N.1
3	KIT	Patrick Hodapp	https://www.chemotion-re	X22562	ComP-20041	https://dx.doi.org/10.14272/DGZQNPCSQDROIH-UHFFFAOYSA-N.1
3	KIT	Patrick Hodapp	https://www.chemotion-re	X16531	ComP-20040	https://dx.doi.org/10.14272/LLQAIDHAAXVYTF-UHFFFAOYSA-N.1
3	KIT	Patrick Hodapp	https://www.chemotion-re	X11509	ComP-10004	https://dx.doi.org/10.14272/LKLAQSQLIWJRIC-UHFFFAOYSA-N.3
3	KIT	Patrick Hodapp	https://www.chemotion-re	X11508	ComP-9632	https://dx.doi.org/10.14272/YRPIXORGESANFA-UHFFFAOYSA-N.5
3	KIT	Patrick Hodapp	https://www.chemotion-re	X7546	ComP-1268	https://dx.doi.org/10.14272/RJQVSFJIBQPCLA-UHFFFAOYSA-N
3	KIT	Patrick Hodapp	https://www.chemotion-re	X10746	ComP-8374	https://dx.doi.org/10.14272/GOUDGRBOWFMVRW-UHFFFAOYSA-N
3	KIT	Patrick Hodapp	https://www.chemotion-re	X8772	ComP-7731	https://dx.doi.org/10.14272/BMIMNRPAEPIYDN-UHFFFAOYSA-N.2
4	KIT	Timo Sehn/SML	https://www.chemotion-re	X18671	ComP-15606	https://dx.doi.org/10.14272/OSMXVZQQWFMTPK-PEPZGXQESA-N.1
4	KIT	Timo Sehn/SML	https://www.chemotion-re	X18668	ComP-15607	https://dx.doi.org/10.14272/BIVYBXKQJWPFNV-PEPZGXQESA-N.1
4	KIT	Timo Sehn/SML	https://www.chemotion-re	X18666	ComP-15611	https://dx.doi.org/10.14272/WPUBWLUSKQXZNQ-SFECMWDFSA-N.1

Datas	Location	author-group	repo sample ID	X-Nummer	molecule archive ID	DOI Repo
4	KIT	Timo Sehn/SML	https://www.chemotion-re	X18669	ComP-15610	https://dx.doi.org/10.14272/UIPWBIZIYFFHOR-XSYHWHKQSA-N.1
4	KIT	Timo Sehn/SML	https://www.chemotion-re	X18670	ComP-15609	https://dx.doi.org/10.14272/OOFBGHCVZDGOMM-PEPZGXQESA-N.1
4	KIT	Timo Sehn/SML	https://www.chemotion-re	X18667	ComP-15608	https://dx.doi.org/10.14272/OUMVYHHZWFPSFH-SFECMWDFSA-N.1
5	Aachen	Sonja Herres-Pawlis/ Fabian Fink	https://www.chemotion-re	X16202	ComP-14248	https://dx.doi.org/10.14272/AMQWWHVPPZUOKP-UHFFFAOYSA-L.1
5	Aachen	Sonja Herres-Pawlis/ Fabian Fink	https://www.chemotion-re	X16272	ComP-14179	https://dx.doi.org/10.14272/IPQNHLBUWYQHDL-UHFFFAOYSA-N.1
5	Aachen	Sonja Herres-Pawlis/ Fabian Fink	https://www.chemotion-re	X16271	ComP-14180	https://dx.doi.org/10.14272/ZZFUHQXQLNLLCA-UHFFFAOYSA-N.1
5	Aachen	Sonja Herres-Pawlis/ Fabian Fink	https://www.chemotion-re	X16207	ComP-14243	https://dx.doi.org/10.14272/QTIQVBQUEFIRBE-UHFFFAOYSA-N.1
5	Aachen	Sonja Herres-Pawlis/ Fabian Fink	https://www.chemotion-re	X16217	ComP-14233	https://dx.doi.org/10.14272/PIKLCVQDBVTHFG-UHFFFAOYSA-M.1
5	Aachen	Sonja Herres-Pawlis/ Regina Schn	https://www.chemotion-re	X16403	ComP-20561	https://dx.doi.org/10.14272/AEZGGXCUYLBMSI-UHFFFAOYSA-N.1
5	Aachen	Sonja Herres-Pawlis/ Regina Schn	https://www.chemotion-re	X16404	ComP-20560	https://dx.doi.org/10.14272/ZLWCJIJDQBYWSZ-UHFFFAOYSA-N.1
5	Aachen	Sonja Herres-Pawlis/ Regina Schn	https://www.chemotion-re	X16251	ComP-20564	https://dx.doi.org/10.14272/CTVCMXWPORVIRI-UHFFFAOYSA-N.1
5	Aachen	Sonja Herres-Pawlis/ Fabian Thor	https://www.chemotion-re	X16401	ComP-20563	https://dx.doi.org/10.14272/NWKTUCNDIPMMKM-TZYAJKAJSA-N.1
5	Aachen	Sonja Herres-Pawlis/ Fabian Thor	https://www.chemotion-re	X16402	ComP-20562	https://dx.doi.org/10.14272/KXRYDZCNUHBCQB-SJWRPRNESA-N.1
5	Aachen	Sonja Herres-Pawlis/ Fabian Thor	https://www.chemotion-re	X16249	ComP-14201	https://dx.doi.org/10.14272/TZHOOUXJQHQALI-UHFFFAOYSA-N.1
5	Aachen	Sonja Herres-Pawlis/ Fabian Thor	https://www.chemotion-re	X16239	ComP-14211	https://dx.doi.org/10.14272/LSGGPBYVWWQPOY-UHFFFAOYSA-N.1
5	Aachen	Sonja Herres-Pawlis/ Fabian Thor	https://www.chemotion-re	X16241	ComP-14209	https://dx.doi.org/10.14272/UFNYJPFRGDSKSE-UHFFFAOYSA-N.1
5	Aachen	Sonja Herres-Pawlis/ Christian Co	https://www.chemotion-re	X16274	ComP-14177	https://dx.doi.org/10.14272/OCSWTCSEJPHWQB-UHFFFAOYSA-N.1
5	Aachen	Sonja Herres-Pawlis/ Fabian Fink	https://www.chemotion-re	X16201	ComP-14249	https://dx.doi.org/10.14272/PFBKJCSJBZOEJW-UHFFFAOYSA-N.1
6	München	Lena Daumann/ Rachel Janßen	https://www.chemotion-re	X23606	ComP-20523	https://dx.doi.org/10.14272/GLVNZYODMKSEPS-ONEGZZNKSA-N.1
6	München	Lena Daumann/ Rachel Janßen	https://www.chemotion-re	X23612	ComP-20514	https://dx.doi.org/10.14272/NDTWZHURUDSPQV-UHFFFAOYSA-N.1
6	München	Lena Daumann/ Rachel Janßen	https://www.chemotion-re	X23603	ComP-20526	https://dx.doi.org/10.14272/HIUDDUFOZHEUFH-UHFFFAOYSA-N.1
6	München	Lena Daumann/ Rachel Janßen	https://www.chemotion-re	X23601	ComP-20527	https://dx.doi.org/10.14272/GJNRAOKPAMBFHT-UHFFFAOYSA-N.1
6	München	Lena Daumann/ Rachel Janßen	https://www.chemotion-re	X23608	ComP-20509	https://dx.doi.org/10.14272/WMFURCICLSTGEJ-UHFFFAOYSA-N.1
6	München	Lena Daumann/ Rachel Janßen	https://www.chemotion-re	X23614	ComP-20513	https://dx.doi.org/10.14272/WMGUYZIVIVLQHB-UHFFFAOYSA-N.1
6	München	Lena Daumann/ Rachel Janßen	https://www.chemotion-re	X23613	ComP-20512	https://dx.doi.org/10.14272/VVKJYIDCUMETHR-UHFFFAOYSA-N.1
6	München	Lena Daumann/ Rachel Janßen	https://www.chemotion-re	X23610	ComP-20515	https://dx.doi.org/10.14272/WPBCYIDVZMJOFN-RIYZIHGNSA-N.1

Datas	Location	author-group	repo sample ID	X-Nummer	molecule archive ID	DOI Repo
6	München	Lena Daumann/ Rachel Janßen	https://www.chemotion-re	X23604	ComP-20525	https://dx.doi.org/10.14272/DCYWQPPHKRQQGQ-UHFFFAOYSA-N.1
6	München	Lena Daumann/ Rachel Janßen	https://www.chemotion-re	X23605	ComP-20524	https://dx.doi.org/10.14272/GMQHYVDUNUVNOT-UHFFFAOYSA-N.1
6	München	Lena Daumann/ Rachel Janßen	https://www.chemotion-re	X23607	ComP-20516	https://dx.doi.org/10.14272/CFUDBJNBUQJVCC-UHFFFAOYSA-N.1
6	München	Lena Daumann/ Rachel Janßen	https://www.chemotion-re	X23611	ComP-20510	https://dx.doi.org/10.14272/OJRNGUZULVWEHY-RIYZIHGNSA-N.1
6	München	Lena Daumann/ Rachel Janßen	https://www.chemotion-re	X23609	ComP-20511	https://dx.doi.org/10.14272/GAPCMYIHVRFISC-UHFFFAOYSA-N.1
7	Greifswald	Andreas Link/ Felix Potlitz	https://www.chemotion-re	X19392	ComP-17455	https://dx.doi.org/10.14272/RAZITXFWTAPVOX-UHFFFAOYSA-N.1
7	Greifswald	Andreas Link/ Felix Potlitz	https://www.chemotion-re	X19329	ComP-17518	https://dx.doi.org/10.14272/ZHULLRQUWLYJHW-UHFFFAOYSA-N.1
7	Greifswald	Andreas Link/ Felix Potlitz	https://www.chemotion-re	X19384	ComP-17463	https://dx.doi.org/10.14272/TWKUAKLZOIABHJ-UHFFFAOYSA-N.1
7	Greifswald	Andreas Link/ Felix Potlitz	https://www.chemotion-re	X19372	ComP-17475	https://dx.doi.org/10.14272/LLVFBEKEDCMPGV-MDWZMJQESA-N.1
7	Greifswald	Andreas Link/ Felix Potlitz	https://www.chemotion-re	X19388	ComP-17459	https://dx.doi.org/10.14272/CSMMSENKKVOGPL-UHFFFAOYSA-N.1
7	Greifswald	Andreas Link/ Felix Potlitz	https://www.chemotion-re	X19311	ComP-17536	https://dx.doi.org/10.14272/UMKRDQFXYJCILQ-UHFFFAOYSA-N.1
7	Greifswald	Andreas Link/ Felix Potlitz	https://www.chemotion-re	X19370	ComP-17477	https://dx.doi.org/10.14272/MZMWXNCBDICVDV-KPKJPENVSA-N.1
8	Kaiserslautern	Georg Manolikakes/ Miro Halaczki	https://www.chemotion-re	X12959	ComP-20776	https://dx.doi.org/10.14272/ZUHSADHMQHGSEJ-GBLZOACLSA-N.1
8	Kaiserslautern	Georg Manolikakes/ Miro Halaczki	https://www.chemotion-re	X12960	ComP-20775	https://dx.doi.org/10.14272/ZUHSADHMQHGSEJ-SMHULIPUSA-N.1
8	Kaiserslautern	Georg Manolikakes/ Miro Halaczki	https://www.chemotion-re	X12961	ComP-20774	https://dx.doi.org/10.14272/FUOUKDJVZAOCOJ-QFWMQHCXSA-N.1
8	Kaiserslautern	Georg Manolikakes/ Miro Halaczki	https://www.chemotion-re	X12962	ComP-20773	https://dx.doi.org/10.14272/FUOUKDJVZAOCOJ-DQMJNTIXSA-N.1
8	Kaiserslautern	Georg Manolikakes/ Miro Halaczki	https://www.chemotion-re	X12957	ComP-20778	https://dx.doi.org/10.14272/BQMXHIRXMSGDCD-AVIJNYRZSA-N.1
8	Kaiserslautern	Georg Manolikakes/ Miro Halaczki	https://www.chemotion-re	X12958	ComP-20777	https://dx.doi.org/10.14272/BQMXHIRXMSGDCD-ZGNKEGEESA-N.1
8	Kaiserslautern	Georg Manolikakes/ Miro Halaczki	https://www.chemotion-re	X12953	ComP-20782	https://dx.doi.org/10.14272/NWCOERFKLVQQOI-IEKZBVHVSA-N.1
8	Kaiserslautern	Georg Manolikakes/ Miro Halaczki	https://www.chemotion-re	X12954	ComP-20781	https://dx.doi.org/10.14272/NWCOERFKLVQQOI-DURYTUKUSA-N.1
8	Kaiserslautern	Georg Manolikakes/ Miro Halaczki	https://www.chemotion-re	X12956	ComP-20784	https://dx.doi.org/10.14272/UWHQATUHQVAVJT-UHFFFAOYSA-N.1
8	Kaiserslautern	Georg Manolikakes/ Miro Halaczki	https://www.chemotion-re	X12955	ComP-20780	https://dx.doi.org/10.14272/MXNBXEFODBPXBQ-UHFFFAOYSA-N.1
8	Kaiserslautern	Georg Manolikakes/ Miro Halaczki	https://www.chemotion-re	X12952	ComP-20783	https://dx.doi.org/10.14272/GJYIWQYBISMEII-UHFFFAOYSA-N.1
8	Kaiserslautern	Georg Manolikakes/ Miro Halaczki	https://www.chemotion-re	X12951	ComP-20784	https://dx.doi.org/10.14272/ZETLVSOYHDFNPZ-UHFFFAOYSA-N.1
9	KIT	Simone Gräßle	https://www.chemotion-re	X6285	ComP-4312	https://dx.doi.org/10.14272/AJUPULBPXRSZPK-UHFFFAOYSA-N.1
9	KIT	Sylvia Vanderheiden	https://www.chemotion-re	X6117	ComP-4100	https://dx.doi.org/10.14272/RCALJRZMLQRHLX-UHFFFAOYSA-N.1

Datas	Location	author-group	repo sample ID	X-Nummer	molecule archive ID	DOI Repo
9	KIT	Simone Gräßle	https://www.chemotion-re	X6235	ComP-4210	https://dx.doi.org/10.14272/RRIKWYIWYSETTC-UHFFFAOYSA-N.1
9	KIT	Sylvia Vanderheiden	https://www.chemotion-re	X9370	ComP-7237	https://dx.doi.org/10.14272/ZNUPABCJWDFYLZ-UHFFFAOYSA-N.1
9	KIT	Simone Gräßle	https://www.chemotion-re	X6236	ComP-4211	https://dx.doi.org/10.14272/LOEMJXFENGAIRJ-UHFFFAOYSA-N.1
9	KIT	Simone Gräßle	https://www.chemotion-re	X9337	ComP-7123	https://dx.doi.org/10.14272/IECVTZNNDJQHMF-UHFFFAOYSA-N.2
9	KIT	Simone Gräßle	https://www.chemotion-re	X6030	ComP-7040	https://dx.doi.org/10.14272/KWUZCAVKPCRJPO-UHFFFAOYSA-N.1
9	KIT	Simone Gräßle	https://www.chemotion-re	X6478	ComP-4597	https://dx.doi.org/10.14272/YEMJQXMSJPHNFJ-UHFFFAOYSA-N.1
9	KIT	Simone Gräßle	https://www.chemotion-re	X6530	ComP-4689	https://dx.doi.org/10.14272/GGFYWXBSNMOKOK-UHFFFAOYSA-N.1
9	KIT	Simone Gräßle	https://www.chemotion-re	X6352	ComP-4493	https://dx.doi.org/10.14272/OEOPVJYUCSQVMJ-UHFFFAOYSA-N.1
9	KIT	Simone Gräßle	https://www.chemotion-re	X6688	ComP-4715	https://dx.doi.org/10.14272/NNXGMQKTMUWZBW-UHFFFAOYSA-N.1
9	KIT	Simone Gräßle	https://www.chemotion-re	X6689	ComP-4718	https://dx.doi.org/10.14272/AZFTWUQDMMNFBC-UHFFFAOYSA-N.1
9	KIT	Simone Gräßle	https://www.chemotion-re	X6691	ComP-4739	https://dx.doi.org/10.14272/AWYKSNJSUDJLBZ-UHFFFAOYSA-N.1
9	KIT	Simone Gräßle	https://www.chemotion-re	X6696	ComP-4795	https://dx.doi.org/10.14272/KMWDVZIQOFYBIA-UHFFFAOYSA-N
9	KIT	Simone Gräßle	https://www.chemotion-re	X6694	ComP-4749	https://dx.doi.org/10.14272/MQTOXMPVDWKAQQ-UHFFFAOYSA-N.1
9	KIT	Simone Gräßle	https://www.chemotion-re	X6485	ComP-4657	https://dx.doi.org/10.14272/QBUUSKFCOZCDNQ-UHFFFAOYSA-N.1
9	KIT	Simone Gräßle	https://www.chemotion-re	X6490	ComP-4642	https://dx.doi.org/10.14272/DFNVJQOYTSHVCU-UHFFFAOYSA-N.1
9	KIT	Simone Gräßle	https://www.chemotion-re	X6499	ComP-4601	https://dx.doi.org/10.14272/YYVNJMDZXBHEAA-UHFFFAOYSA-N.1
9	KIT	Simone Gräßle	https://www.chemotion-re	X6487	ComP-4654	https://dx.doi.org/10.14272/MHUTZKFXGAZXEC-UHFFFAOYSA-N.1
9	KIT	Simone Gräßle	https://www.chemotion-re	X6695	ComP-4751	https://dx.doi.org/10.14272/PQNMDYKEGIJCTR-UHFFFAOYSA-N.1
9	KIT	Simone Gräßle	https://www.chemotion-re	X8793	ComP-6648	https://dx.doi.org/10.14272/KGPPSSIWSDMFRL-UHFFFAOYSA-N.2
9	KIT	Simone Gräßle	https://www.chemotion-re	X6381	ComP-4491	https://dx.doi.org/10.14272/YOTWMBDNLPNRKE-UHFFFAOYSA-N.1
9	KIT	Simone Gräßle	https://www.chemotion-re	X6320	ComP-4561	https://dx.doi.org/10.14272/ADRCREMWPUFGDU-UHFFFAOYSA-N.1
9	KIT	Simone Gräßle	https://www.chemotion-re	X6527	ComP-4690	https://dx.doi.org/10.14272/FHJRKGXJBXPBGA-UHFFFAOYSA-N.1
9	KIT	Simone Gräßle	https://www.chemotion-re	X6370	ComP-4553	https://dx.doi.org/10.14272/HYKGLCSXVAAXNC-UHFFFAOYSA-N.1
9	KIT	Simone Gräßle	https://www.chemotion-re	X6686	ComP-4674	https://dx.doi.org/10.14272/XBHOUXSGHYZCNH-UHFFFAOYSA-N.1
9	KIT	Simone Gräßle	https://www.chemotion-re	X6528	ComP-4714	https://dx.doi.org/10.14272/NAUXHTYTQIQZJI-UHFFFAOYSA-N.1
9	KIT	Simone Gräßle	https://www.chemotion-re	X7324	ComP-8663	https://dx.doi.org/10.14272/ODMDLCWSMSFWCW-UHFFFAOYSA-N.1

Datas	Location	author-group	repo sample ID	X-Nummer	molecule archive ID	DOI Repo
9	KIT	Simone Gräßle	https://www.chemotion-re	X11158	ComP-8991	https://dx.doi.org/10.14272/JIWVTDBBMGSTFI-UHFFFAOYSA-N
9	KIT	Simone Gräßle	https://www.chemotion-re	X11157	ComP-8992	https://dx.doi.org/10.14272/MVVGSPCXHRFDDR-UHFFFAOYSA-N.1
9	KIT	Simone Gräßle	https://www.chemotion-re	X6498	ComP-4602	https://dx.doi.org/10.14272/KEJBDGQLLLLBGE-UHFFFAOYSA-N.1
9	KIT	Simone Gräßle	https://www.chemotion-re	X6488	ComP-4653	https://dx.doi.org/10.14272/FOURLJNIFQPOFD-UHFFFAOYSA-N.1
9	KIT	Simone Gräßle	https://www.chemotion-re	X9556	ComP-7393	https://dx.doi.org/10.14272/MWIDLEVLPMTJDU-UHFFFAOYSA-N.1
9	KIT	Simone Gräßle	https://www.chemotion-re	X9561	ComP-7402	https://dx.doi.org/10.14272/BQLDYARBQZEEIU-UHFFFAOYSA-N.3
9	KIT	Simone Gräßle	https://www.chemotion-re	X6328	ComP-4498	https://dx.doi.org/10.14272/ZYCYJFGYAPFPFF-UHFFFAOYSA-N.1
9	KIT	Simone Gräßle	https://www.chemotion-re	X6484	ComP-4656	https://dx.doi.org/10.14272/QNPXQKVFKDIPED-UHFFFAOYSA-N.1
9	KIT	Simone Gräßle	https://www.chemotion-re	X6318	ComP-4556	https://dx.doi.org/10.14272/JTJKNVJLPWBSFH-UHFFFAOYSA-N.1
9	KIT	Simone Gräßle	https://www.chemotion-re	X6685	ComP-4673	https://dx.doi.org/10.14272/SVTDXKPYBJXBJR-UHFFFAOYSA-N.1
9	KIT	Simone Gräßle	https://www.chemotion-re	X6687	ComP-4716	https://dx.doi.org/10.14272/AJKDFBCTGMJQSV-UHFFFAOYSA-N.1
9	KIT	Simone Gräßle	https://www.chemotion-re	X6683	ComP-4659	https://dx.doi.org/10.14272/XNPQNKNZBZIPFJ-UHFFFAOYSA-N.1
9	KIT	Simone Gräßle	https://www.chemotion-re	X6493	ComP-4613	https://dx.doi.org/10.14272/VKEFWPZGDKNHPJ-UHFFFAOYSA-N.1
9	KIT	Simone Gräßle	https://www.chemotion-re	X6480	ComP-4596	https://dx.doi.org/10.14272/OILOLZJYRKDNOH-UHFFFAOYSA-N.1
9	KIT	Simone Gräßle	https://www.chemotion-re	X6529	ComP-4694	https://dx.doi.org/10.14272/GMLILFMFNDCNOD-UHFFFAOYSA-N.1
9	KIT	Simone Gräßle	https://www.chemotion-re	X6361	ComP-4500	https://dx.doi.org/10.14272/FYOMFFHHDXMMIU-UHFFFAOYSA-N.1
9	KIT	Simone Gräßle	https://www.chemotion-re	X6684	ComP-4672	https://dx.doi.org/10.14272/PDVSHXSSPVXJNH-UHFFFAOYSA-N.1
9	KIT	Simone Gräßle	https://www.chemotion-re	X6690	ComP-4720	https://dx.doi.org/10.14272/NISURZRTIYSNSA-UHFFFAOYSA-N.1
9	KIT	Simone Gräßle	https://www.chemotion-re	X11219	ComP-9009	https://dx.doi.org/10.14272/JFDRLDCIZJRMON-UHFFFAOYSA-N.1
9	KIT	Simone Gräßle	https://www.chemotion-re	X8787	ComP-6642	https://dx.doi.org/10.14272/NOWQHDRUEYRYJH-UHFFFAOYSA-N.2
9	KIT	Simone Gräßle	https://www.chemotion-re	X6682	ComP-4658	https://dx.doi.org/10.14272/VWIUYPPDMZVIQO-UHFFFAOYSA-N.1
9	KIT	Simone Gräßle	https://www.chemotion-re	X8790	ComP-6629	https://dx.doi.org/10.14272/HVPGYAGVCOFPJH-UHFFFAOYSA-N.2
9	KIT	Simone Gräßle	https://www.chemotion-re	X6388	ComP-4506	https://dx.doi.org/10.14272/QAQOBOMSEMAHEE-UHFFFAOYSA-N.1
9	KIT	Simone Gräßle	https://www.chemotion-re	X8791	ComP-6647	https://dx.doi.org/10.14272/ORVHFNYZXLMSAN-UHFFFAOYSA-N.1
9	KIT	Simone Gräßle	https://www.chemotion-re	X5956	ComP-3080	https://dx.doi.org/10.14272/QBGRAELMPIMNON-UHFFFAOYSA-N.1
9	KIT	Simone Gräßle	https://www.chemotion-re	X6461	ComP-4562	https://dx.doi.org/10.14272/ZRKUQAXOMUSPEH-UHFFFAOYSA-N.1

Datas	Location	author-group	repo sample ID	X-Nummer	molecule archive ID	DOI Repo
9	KIT	Simone Gräßle	https://www.chemotion-re	X6500	ComP-4600	https://dx.doi.org/10.14272/FMWJFHMFGDMYAB-UHFFFAOYSA-N.1
9	KIT	Simone Gräßle	https://www.chemotion-re	X6353	ComP-4555	https://dx.doi.org/10.14272/ZXNGWGZCPWDZJD-UHFFFAOYSA-N.1
9	KIT	Simone Gräßle	https://www.chemotion-re	X4165	ComP-1674	https://dx.doi.org/10.14272/VUMZNLOQJGKGNE-UHFFFAOYSA-N.1
10	KIT-IFG	Lena Pilz/Manuel Tsotsalas	https://www.chemotion-re	X24401	ComP-20990	https://dx.doi.org/10.14272/JMHFTDFPRQWUAN-UHFFFAOYSA-H.12
10	KIT-IFG	Lena Pilz/Manuel Tsotsalas	https://www.chemotion-re	X24402	ComP-20989	https://dx.doi.org/10.14272/JMHFTDFPRQWUAN-UHFFFAOYSA-H.1
10	KIT-IFG	Lena Pilz/Manuel Tsotsalas	https://www.chemotion-re	X24403	ComP-20988	https://dx.doi.org/10.14272/JMHFTDFPRQWUAN-UHFFFAOYSA-H.13
10	KIT-IFG	Lena Pilz/Manuel Tsotsalas	https://www.chemotion-re	X24404	ComP-20987	https://dx.doi.org/10.14272/JMHFTDFPRQWUAN-UHFFFAOYSA-H.22
10	KIT-IFG	Lena Pilz/Manuel Tsotsalas	https://www.chemotion-re	X24405	ComP-20986	https://dx.doi.org/10.14272/JMHFTDFPRQWUAN-UHFFFAOYSA-H.14
10	KIT-IFG	Lena Pilz/Manuel Tsotsalas	https://www.chemotion-re	X24406	ComP-20985	https://dx.doi.org/10.14272/JMHFTDFPRQWUAN-UHFFFAOYSA-H.23
10	KIT-IFG	Lena Pilz/Manuel Tsotsalas	https://www.chemotion-re	X24407	ComP-20984	https://dx.doi.org/10.14272/JMHFTDFPRQWUAN-UHFFFAOYSA-H.15
10	KIT-IFG	Lena Pilz/Manuel Tsotsalas	https://www.chemotion-re	X24408	ComP-20983	https://dx.doi.org/10.14272/JMHFTDFPRQWUAN-UHFFFAOYSA-H.24
10	KIT-IFG	Lena Pilz/Manuel Tsotsalas	https://www.chemotion-re	X24409	ComP-20982	https://dx.doi.org/10.14272/JMHFTDFPRQWUAN-UHFFFAOYSA-H.25
10	KIT-IFG	Lena Pilz/Manuel Tsotsalas	https://www.chemotion-re	X24410	ComP-20981	https://dx.doi.org/10.14272/JMHFTDFPRQWUAN-UHFFFAOYSA-H.16
10	KIT-IFG	Lena Pilz/Manuel Tsotsalas	https://www.chemotion-re	X24411	ComP-20980	https://dx.doi.org/10.14272/JMHFTDFPRQWUAN-UHFFFAOYSA-H.26
10	KIT-IFG	Lena Pilz/Manuel Tsotsalas	https://www.chemotion-re	X24412	ComP-20979	https://dx.doi.org/10.14272/JMHFTDFPRQWUAN-UHFFFAOYSA-H.17
10	KIT-IFG	Lena Pilz/Manuel Tsotsalas	https://www.chemotion-re	X24413	ComP-20978	https://dx.doi.org/10.14272/JMHFTDFPRQWUAN-UHFFFAOYSA-H.27
10	KIT-IFG	Lena Pilz/Manuel Tsotsalas	https://www.chemotion-re	X24414	ComP-20977	https://dx.doi.org/10.14272/JMHFTDFPRQWUAN-UHFFFAOYSA-H.18
10	KIT-IFG	Lena Pilz/Manuel Tsotsalas	https://www.chemotion-re	X24415	ComP-20976	https://dx.doi.org/10.14272/JMHFTDFPRQWUAN-UHFFFAOYSA-H.28
10	KIT-IFG	Lena Pilz/Manuel Tsotsalas	https://www.chemotion-re	X24416	ComP-20975	https://dx.doi.org/10.14272/JMHFTDFPRQWUAN-UHFFFAOYSA-H.19
10	KIT-IFG	Lena Pilz/Manuel Tsotsalas	https://www.chemotion-re	X24417	ComP-20974	https://dx.doi.org/10.14272/JMHFTDFPRQWUAN-UHFFFAOYSA-H.2
10	KIT-IFG	Lena Pilz/Manuel Tsotsalas	https://www.chemotion-re	X24418	ComP-20973	https://dx.doi.org/10.14272/JMHFTDFPRQWUAN-UHFFFAOYSA-H.29
10	KIT-IFG	Lena Pilz/Manuel Tsotsalas	https://www.chemotion-re	X24419	ComP-20972	https://dx.doi.org/10.14272/JMHFTDFPRQWUAN-UHFFFAOYSA-H.20
10	KIT-IFG	Lena Pilz/Manuel Tsotsalas	https://www.chemotion-re	X24420	ComP-20971	https://dx.doi.org/10.14272/JMHFTDFPRQWUAN-UHFFFAOYSA-H.3
10	KIT-IFG	Lena Pilz/Manuel Tsotsalas	https://www.chemotion-re	X24421	ComP-20970	https://dx.doi.org/10.14272/JMHFTDFPRQWUAN-UHFFFAOYSA-H.30
10	KIT-IFG	Lena Pilz/Manuel Tsotsalas	https://www.chemotion-re	X24422	ComP-20969	https://dx.doi.org/10.14272/JMHFTDFPRQWUAN-UHFFFAOYSA-H.21
10	KIT-IFG	Lena Pilz/Manuel Tsotsalas	https://www.chemotion-re	X24423	ComP-20968	https://dx.doi.org/10.14272/JMHFTDFPRQWUAN-UHFFFAOYSA-H.5

Datas	Location	author-group	repo sample ID	X-Nummer	molecule archive ID	DOI Repo
10	KIT-IFG	Lena Pilz/Manuel Tsotsalas	https://www.chemotion-re	X24424	ComP-20967	https://dx.doi.org/10.14272/JMHFTDFPRQWUAN-UHFFFAOYSA-H.4
10	KIT-IFG	Lena Pilz/Manuel Tsotsalas	https://www.chemotion-re	X24425	ComP-20966	https://dx.doi.org/10.14272/JMHFTDFPRQWUAN-UHFFFAOYSA-H.6
10	KIT-IFG	Lena Pilz/Manuel Tsotsalas	https://www.chemotion-re	X24426	ComP-20965	https://dx.doi.org/10.14272/JMHFTDFPRQWUAN-UHFFFAOYSA-H.7
10	KIT-IFG	Lena Pilz/Manuel Tsotsalas	https://www.chemotion-re	X24427	ComP-20964	https://dx.doi.org/10.14272/JMHFTDFPRQWUAN-UHFFFAOYSA-H.8
10	KIT-IFG	Lena Pilz/Manuel Tsotsalas	https://www.chemotion-re	X24428	ComP-20963	https://dx.doi.org/10.14272/JMHFTDFPRQWUAN-UHFFFAOYSA-H.9
10	KIT-IFG	Lena Pilz/Manuel Tsotsalas	https://www.chemotion-re	X24429	ComP-20962	https://dx.doi.org/10.14272/JMHFTDFPRQWUAN-UHFFFAOYSA-H.10
10	KIT-IFG	Lena Pilz/Manuel Tsotsalas	https://www.chemotion-re	X24430	ComP-20961	https://dx.doi.org/10.14272/JMHFTDFPRQWUAN-UHFFFAOYSA-H.11
11	Mainz	Till Opatz	https://www.chemotion-re	X20572	ComP-17839	https://dx.doi.org/10.14272/LBLPBDUCKUHMIA-UHFFFAOYSA-N.1
11	Mainz	Till Opatz	https://www.chemotion-re	X20513	ComP-17898	https://dx.doi.org/10.14272/CBWRQOOQZVGWJP-UHFFFAOYSA-N.1
11	Mainz	Till Opatz	https://www.chemotion-re	X20509	ComP-17902	https://dx.doi.org/10.14272/FREVBOAFRVSAIJ-UHFFFAOYSA-N.1
11	Mainz	Till Opatz	https://www.chemotion-re	X20512	ComP-17899	https://dx.doi.org/10.14272/PVXVETMDFUEGCN-UHFFFAOYSA-N.1
11	Mainz	Till Opatz	https://www.chemotion-re	X20510	ComP-20928	https://dx.doi.org/10.14272/PCLDLWKAKMKZFX-UHFFFAOYSA-N.1
12	Ilmenau	Eric Täuscher/ Emma Freiberger	https://www.chemotion-re	X23792	ComP-20998	https://dx.doi.org/10.14272/FMENTXWLFCBOIS-UDWIEESQSA-N.1
12	Ilmenau	Eric Täuscher/ Emma Freiberger	https://www.chemotion-re	X23791	ComP-20999	https://dx.doi.org/10.14272/DPNBPQMTXJNKBM-UHFFFAOYSA-N
